# Impact of the Mid-Pleistocene Revolution and Anthropogenic Factors on the Dispersion of Asian Black-Spined Toads (*Duttaphrynus melanostictus*)

**DOI:** 10.3390/ani10071157

**Published:** 2020-07-08

**Authors:** Siti N. Othman, Yi-Huey Chen, Ming-Feng Chuang, Desiree Andersen, Yikweon Jang, Amaël Borzée

**Affiliations:** 1 Department of Life Sciences and Division of EcoScience, Ewha Womans University, Seoul 03760, Korea; dy.othman@gmail.com (S.N.O.); adammfc@gmail.com (M.-F.C.); desireeka93@hotmail.com (D.A.); jangy@ewha.ac.kr (Y.J.); 2 Department of Life Science, Chinese Culture University, Taipei 11114, Taiwan; yihueyc@gmail.com; 3 Department of Life Sciences, National Chung Hsing University, Taichung 40227, Taiwan; 4 Laboratory of Animal Behaviour and Conservation, College of Biology and the Environment, Nanjing Forestry University, Nanjing 210037, China

**Keywords:** indomalayan realm, pleistocene glaciations, holocene, bayesian inference, anthropogenic impact

## Abstract

**Simple Summary:**

Three distinct lineages of *Duttaphrynus melanostictus*, the Asian black-spined toad, are present in Southeast Asia. However, divergence times, dispersion mechanisms and colonisation processes are still unknown. In the present study, molecular dating based on mitochondrial DNA sequences demonstrated that *D. melanostictus* expanded into Eastern Indomalaya following the Quaternary glaciation and colonised new landscapes during the Last Glacial Maximum. Subsequent to natural colonisation of landscapes, we found human-induced dispersal into regions such as in Taiwan, Southern Sundaic and Wallacea, temporally matching with prehistoric human settlements. We provide comprehensive dispersal pathways and mechanisms of *D. melanostictus* to the Eastern Indomalayan realm, thus solving the climate-driven question relevant to the species distribution in the Southeast Asia.

**Abstract:**

Divergence-time estimation critically improves the understanding of biogeography processes underlying the distribution of species, especially when fossil data is not available. We hypothesise that the Asian black-spined toad, *Duttaphrynus melanostictus*, expanded into the Eastern Indomalaya following the Quaternary glaciations with the subsequent colonisation of new landscapes during the Last Glacial Maximum. Divergence dating inferred from 364 sequences of mitochondrial *tRNAGly ND3* supported the emergence of a common ancestor to the three *D. melanostictus* clades around 1.85 (±0.77) Ma, matching with the Lower to Mid-Pleistocene transition. *Duttaphrynus melanostictus* then dispersed into Southeast Asia from the central Indo-Pacific and became isolated in the Southern Sundaic and Wallacea regions 1.43 (±0.10) Ma through vicariance as a result of sea level oscillations. The clade on the Southeast Asian mainland then colonised the peninsula from Myanmar to Vietnam and expanded towards Southeastern China at the end of the Mid-Pleistocene Revolution 0.84 (±0.32) Ma. Population dynamics further highlight an expansion of the Southeast Asian mainland population towards Taiwan, the Northeastern edge of the species’ range after the last interglacial, and during the emergence of the Holocene human settlements around 7000 BP. Thus, the current divergence of *D. melanostictus* into three segregated clades was mostly shaped by Quaternary glaciations, followed by natural dispersion events over land bridges and accelerated by anthropogenic activities.

## 1. Introduction

The Pleistocene was punctuated by climatic oscillations resulting in the repeated decrease and increase of global temperatures [1]. Consequently, the Pleistocene climatic oscillations have influenced faunal population structures and led to species-complex forming as a result of local adaptations [2]. In the context of amphibians, quaternary climatic oscillations in the Western Palearctic gave rise to new species such as the Northern American toad *Anaxyrus punctatus* [3] and the European common toad *Bufo bufo* [4]. In Asia, variation in atmospheric temperature, precipitation and humidity resulted in changes in the water levels of freshwater bodies between the late Pleistocene and the last interglacial, as exemplified in reconstructions of the paleoclimates of the Qinghai-Tibetan plateau [5]. Variations in atmospheric temperatures also led to the formation of several refugia for populations of Southwest species in Northern and Eastern Asia [6,7]. For instance, these paleoclimatic events resulted in the divergence of Yunnan slow frogs, *Nanorana* spp. lineages, with *Nanorana parkeri* distributed on the Tibetan Plateau [8] and *N. pleskei* distributed further east of the plateau [9]. Also linked to refugia isolations during the Quaternary glaciation, the Mongolian toad, *Pseudepidalea raddei*, segregated into two lineages, one in western-central China and a second one further east [10]. Further north, the Quaternary ice age was prevalent in the development of the complex genetic structure of *B. gargarizans* [11].

Extensive research on the implications of the Quaternary glaciation are generally limited to North America [12], Europe [13] and Australia [14]. The situation in Southeast Asia is not as clearly resolved despite the determination of phylogenetic relationships for numerous amphibian groups [15,16,17,18,19,20]. Among the resolved climate-driven phylogenies, the Four-lined treefrog, *Polypedates leucomystax* (Anura: Rhacophoridae), was shown to display a hierarchical structure highlighting population expansions across islands, such as the Philippines, followed by recent human-induced dispersal [21]. In addition, three bufonids (*Pelophryne signata*, *Ingerophrynus parvus* and *Leptophryne borbonica*) show strong patterns of allopatric divergence likely linked to the rise of sea level and isolation on islands [22].

Following the Pleistocene glaciations, climatic oscillations between 1.2 and 0.6 Ma drastically shifted in periodicity and duration. This resulted in a different pattern in glacial and interglacial cycles, dubbed the Mid-Pleistocene Revolution (MPR; [23]). The variations in ice volume during the MPR resulted in fluctuations of the sea level in Southeast Asia, alternatively facilitating and limiting connectivity, thus resulting in the general diversification of Indomalayan vertebrates [24]. Following the MPR, the late Quaternary witnessed lower rainfalls, longer dry seasons and lower sea levels in Southeast Asia. These variations exposed the continental Sunda Shelf, connecting the landmasses of Vietnam, Borneo and Java [25,26]. This land bridge facilitated connectivity of populations through faunal migration across the shelf and towards newly emerged regions such as Sundaland, as evidenced from the estimated age of plant microfossils and sedimentary rocks [27,28,29].

Two different hypotheses explain the simultaneous dispersal pathways and colonisation routes used by vertebrates to enter the Eastern Indomalayan realm during the early Pleistocene (~2.6 Ma). (1) Asian vertebrates dispersed from the southern Southeast Asian mainland to the northern Sundaic and then crossed the Sundaic to reach Wallace’s Line. (2) Some species from western and eastern Southeast Asia dispersed along an eastern route via the Philippines and then migrated southward along land bridges into the Sundaic, Wallacea and current Sulawesi [30,31]. Later, the fauna dispersing along the western and eastern pathways may have created secondary contact zones in the Southern Sundaic (Upper Pleistocene, 1.8 Ma) [32]. This point is, however, contentious, with the eastern and western dispersion routes potentially not linking in Southern Sundaic due to tectonic movement [33], raising more questions on the impact of extreme climate transition on distribution pathways [34].

Later on, during the last interglacial between the late Pleistocene and Mid-Holocene (18,000 to 5000 BP), the Last Glacial Maximum (LGM) altered the vegetation of the exposed the Sunda Shelf (LGM; 30,000 to 23,000 BP; [35,36,37]). Then, deglaciation resulted in the flooding of the Sunda Shelf and the Indonesian region became wetter during the Mid-Holocene (11,600 BP; [38]), shaping finer details of the current genetic structure of species in Southeast Asia through habitat change [39] and isolation [40].

Genetic data and paleobiogeography studies suggest a subsequent pattern of displacement for some amphibians reaching the contiguous landscapes of Southeast Asia from the Western Ghats, through the Myanmar-Malay Peninsula (Early Neogene; 23.0 Ma; [41,42]). However, the period of arrival of *Duttaphrynus melanostictus*, the focal species of this project, to the Eastern Indomalayan realm has not yet been determined, partially due to the absence of fossil records [43]. The closest fossil, a common ancestor to all extant *D. melanostictus*, was excavated from the late Pleistocene and Holocene Kurnool Caves in South India (~20,000 BP; [44,45]). Its estimated age is concurrent to the phase of severe aridity in South Asia that led to the extinction of small vertebrates (LGM; [46]).

Since the development of trade by humans, *D. melanostictus* unintentionally benefited from being locked into shipping containers and became unwilling hitchhikers between seaports and areas outside of it range [47]. The oldest documented report of *D. melanostictus* invasion in the Anthropocene through human-induced dispersal is in Bali, Indonesia [48]. As a result, of hitchhiking, the species is now invasive in Madagascar [49], East Timor [50] and it has been captured in Northern Queensland and New South Wales in Australia [51]. Likewise, the native status of *D. melanostictus* on islands bordering its range, such as Taiwan [52], is questionable and needs confirmation.

The main goals of this study were to (1) assess the impact of past geological events on the genetic structure and distribution of *D. melanostictus* in the Eastern Indomalayan realm; (2) examine the colonisation mechanisms during the MPR and LGM; and (3) assess the impacts of anthropogenic activities on the current distribution of *D. melanostictus*.

## 2. Materials and Methods

### 2.1. Taxa Sampling and DNA Extraction

The Asian black-spined toad (*Duttaphrynus melanostictus*) is widespread throughout the Indomalayan realm and listed as being of Least Concern under the IUCN red list of endangered species due to its large population sizes [53]. To trace the origin and dispersal routes to Taiwan, we collected molecular samples of *D. melanostictus* in Yangmingshan National Park (*n* = 17) and Tunghai University (*n* = 3). For molecular sampling, all individuals were orally swabbed (cotton-tipped swab; 16H22, Medical Wire; Corsham, UK) and released at the point of capture. All samples were stored at −20 °C until DNA extraction. Total genomic DNA was extracted using Qiagen DNeasy kit (QIAGEN Group, Hilden, Germany) according to the manufacturer’s protocol. To base our analyses on an extensive and balanced sampling, we also acquired 345 matching sequences for the mtDNA *tRNAGly ND3* fragment and 116 sequences for the nuDNA *SOX9* fragment from Genbank. These sequences generally arise from the major taxonomy studies conducted by Wogan et al. (2016) [54] and Vences et al. (2017) [55].

### 2.2. PCR Amplification and DNA Sequencing

For all samples, we amplified a 325 bp fragment of *tRNAGly–ND3* mitochondrial genes (mtDNA) using the primers obtained from Vences et al. (2017) [55] and Wogan et al. (2016) [54], with the original source retrieved from Stuart et al. (2006) [56]. We also amplified 590 bp of the nuclear gene *SOX9* using the primers described by Wogan et al. (2016) [54]. We performed all Polymerase Chain Reactions (PCR) amplification in 20 μL of total reaction, each containing approximately 50 ng of genomic DNA, 1 μL Ex taq (5 units/μL; HR001A, Takara; Shiga, Japan), 1.6 μL of DNTP Mix (10 mM, Takara; Shiga, Japan), 1 μL of both forward and reverse primer (10 μM) and 1.5 μL of MgCl_2_ (2.5 mM). PCR amplifications for *tRNAGly–ND3* were conducted at an initial denaturation temperature of 95 °C for 5 min, followed by 35 cycles such as 94 °C for 30 s, 55 °C for 30 s and 72 °C for 60 s, followed by a final elongation at 72 °C for 10 min. *SOX9* was amplified under the same initial denaturation variables and for 35 cycles as well, but with the following thermal profile: 94 °C for 45 s, 50 °C for 30 s, 72 °C for 60 s and a final elongation at 72 °C for 10 min. All amplifications were carried with a thermocycler SimpliAmp™ Thermal Cycler (A24812, Applied Biosystems; Foster City, CA, USA). All PCR amplicons were purified and sequenced for both forward and reverse strands on an ABI platform (Cosmo Genetech Company Co., Ltd.; Seoul, Korea).

### 2.3. Haplotype Network and Phylogenetic Analyses

**Figure 1:**
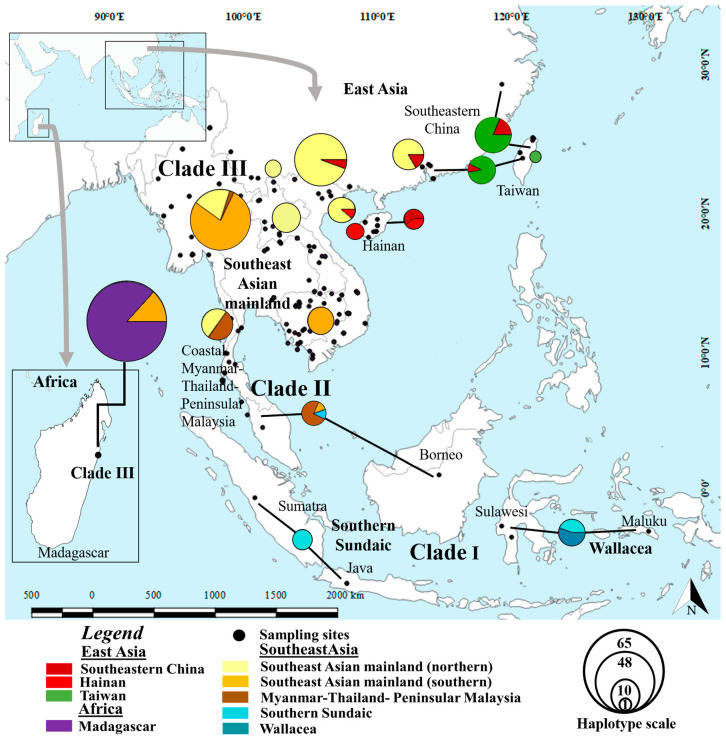
Haplotype networks built from 363 *Duttaphrynus melanostictus* individuals inferred from 336 bp of *tRNAGly ND3* mtDNA fragments. The size of the pie-charts is proportional to the number of samples represented by each haplotype. The symbols and colours used in the legend correspond with those on the haplotype network.

We assessed the general pattern of genetic diversity of 360 *D. melanostictus* individuals (*n* = 341 sampled from Genbank, *n* = 19 sampled in this study) by calculating the haplotype number (*N*) and haplotype diversity (*h*) from 332 bp of the *tRNAGly–ND3* mtDNA fragment. We segregated individuals into 10 populations based on the relative distance between each population (> 100 km), whereby the geographical regions and landscape factors were comprised of: (1) Southeast Asian mainland: northern parts of mainland populations present in Northern Myanmar, Laos, Northern Vietnam. (2) Southeast Asian mainland: south of mainland populations present in Southern Vietnam and Cambodia. (3) Coastal Southeast Asia: Southern Myanmar, Southern Thailand and Peninsular Malaysia. (4) Continental Southeast Asia: Northern Sundaic and Borneo. (5) Southern Sundaic; Sumatra and Java. (6) Wallacea: Sulawesi and Maluku. (7) East Asian mainland: Southeastern China. (8) East Asian island: Hainan. (9) Northeast Asian island: Taiwan. (10) Invasive population in Madagascar (see colour-coded groups in Figure 1). We computed a haplotype network using DNAsp v.5.0 [57] based on maximum parsimony and using the median joining approach [58]. The network estimation was constructed on a 95% probability limit with POPart v.1.7 [59]. We selected a median joining network as it is ideal to infer the evolutionary relationship for intraspecific data [60].

**Figure 2:**
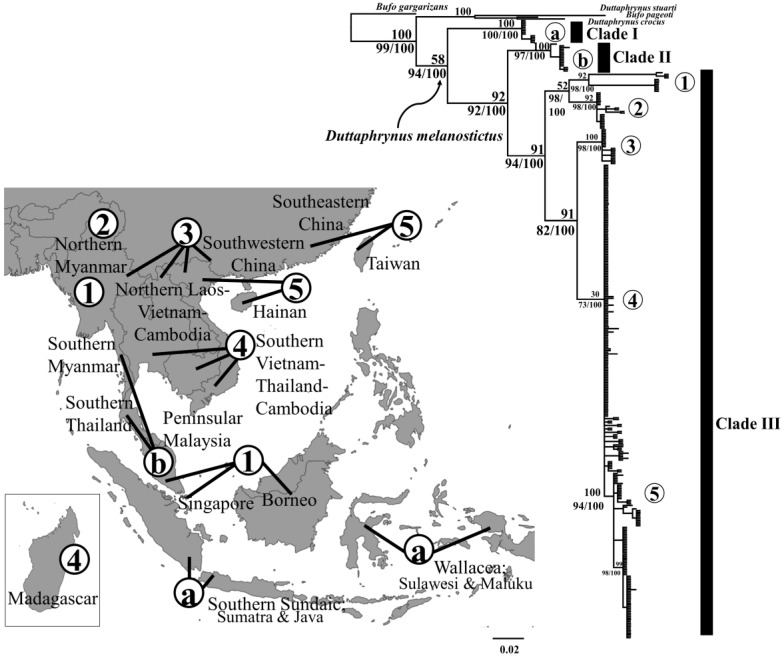
Bayesian Inference (BI) and Maximum Likelihood (ML) tree based on a 324 bp of the mitochondrial gene fragment *trRNA-Gly ND3* extracted from 368 *Duttaphrynus melanostictus* individuals. Samples originate from the Eastern Indomalayan, Eastern Palearctic and Affrogate realm, with *Duttaphrynus stuarti, Duttaphrynus crocus, Bufo pageoti* and *Bufo gargarizans* used as outgroups. Posterior probability (>50%) and bootstrap support (>70/100) are indicated at nodes in the order of ML (1000) replicates.

Phylogenetic trees provide information on population structure and evolutionary relationships between clades and their recent common ancestors. Thus, we reconstructed phylogenetic trees based on 332 bp of the *tRNAGly–ND3* mtDNA fragments (*n* = 364). We also added four sequences as outgroups (Figure 2): *Duttaphrynus crocus* (accession numbers: KU183331)*, Duttaphrynus stuarti* (KU183489)*, Bufo pageoti* (KU183330) and *Bufo gargarizans* (KU183323). Data partitioning was determined using Partition Finder v.2.1.1. [61]. Partition finder recovered six partitions for our fragments based on different codon positions (*tRNAGly* = 1–51 bp; *ND3 =* 51–332 bp) and computed the best substitution model based on a Bayesian Information Criterion (BIC) with a greedy algorithm fitting the setting of MrBayes v.3.1.2 [62]. The best determined models were K80, JC, JC for each of the three partition of *tRNAGly* fragment and GTR+I, K80+G, HKY+I for each partition of the *ND3* fragment. Thus, we reconstructed a Bayesian Inference (BI) tree with MrBayes v.3.1.2 [62] following all the models determined above. To build the tree, we computed two independent Bayesian runs for four Markov Chain Monte Carlo (MCMC) chains over 170,000,000 generations, with a sampling interval of 1000 generations and a ‘burn-in’ corresponding to the first 25% of the tree. Convergence of the parallel runs was confirmed by comparing the split frequencies of standard deviations [63]. The standard deviation of split frequencies for the four Bayesian runs was 0.0095 and the trace plots of clade probabilities were stationary when viewed on Tracer v.1.7.1 [64]. This suggests that the four runs in each analysis had sufficiently converged and that topologies were sampled in proportion to their true posterior probability distribution. Finally, a Maximum Likelihood (ML) tree was also reconstructed in order to get a congruent tree topology under a BI approach. The ML tree was run with RAxML v.0.6.0 [65] and IQTree [66].

### 2.4. Molecular Dating

To date the divergence between clades, we used the 332 bp of the *trNAGly–ND3* mtDNA fragments. We assigned 300 *D. melanostictus* to the ingroup and four *Bufo gargarizans* sequences as outgroup. For this analysis, we excluded the invasive population in Madagascar (*n* = 68), as it is a contemporary event and not relevant to this test [67]. First, we tested the global clock hypothesis using a Maximum Likelihood approach in PAUP v.4.0 [68]. This hypothesis assumes that the rate of evolution among all branches of a phylogenetic tree is the same. PAUP v.4.0 produced a single most probable ML tree out of 305 parsimonious trees, with a likelihood (−lnL) value for the clock model equal to 2089.13 and with an estimated ratio number of transitions to transversions for a pair of sequences (Ti/tv) equal to 4.84. We also ran a local clock model test to check the presence of significant constraint in the subtrees using HyPhy v.2.2.4 [69]. We obtained a –lnL value of 31,700, with the local clock model hypothesis rejected in our tree (*p* > 0.001). These tests indicated that a strict molecular clock was appropriate for our phylogenetic tree and rejected the local clock model. Then, we estimated the divergence date for our mtDNA dataset using BEAST v.2.4.8 [70]. *Bufo gargarizans* served as the best proxy for most recent common ancestor (TRMCA) for *D. melanostictus* with well-documented molecular dating analysis used as reference. We set priors for a calibration point based on the Qinghai-Tibetan Plateau uplift that caused the split between highland and lowland *B. gargarizans* around 3.02 Ma [71,72]. We then computed the pairwise divergence using DiveIn webserver [73] with four rates categories of a discrete gamma model, a sufficient number to analyse a site-specific mutation rate for a single gene [74]. From this computation, we obtained a gamma shape value of 0.328. We then used this value to calculate the pairwise diversity using the formula T = (dxy/mu)/2 where (T) represents divergence in Ma for a given lineage and (mu) represents the mutation rates. For *tRNAGly–ND3*, this resulted in a substitution rates of 0.049 per 3.02 Ma, or an equivalent to 1.6% divergence per million years.

As we failed to reject the molecular clock hypothesis, the HKY substitution model and a strict clock model were selected for the best fitting model with 0.049 clock rates. Using ‘birth-death speciation’ as tree-prior, we implemented a log normal distribution with the mean value of TRMCA of 3.02 Ma, with a standard deviation of 0.5 substitutions per site (3.522–2.52 Ma). The prior of the log normal distribution was selected based on the convergence of results we obtained for all effective sample sizes (ESS) after revising all tested priors and calibrations. We then performed two independent runs of 10,000,000 MCMC chain length separately with a 10% burn-in [63]. The posterior samples for all runs were combined with Log combiner v.1.6.1 [75], a program built-in with BEAST to combine different independent log runs, before diagnosing the convergence status with Tracer v.1.7.1 [75].

**Figure 3:**
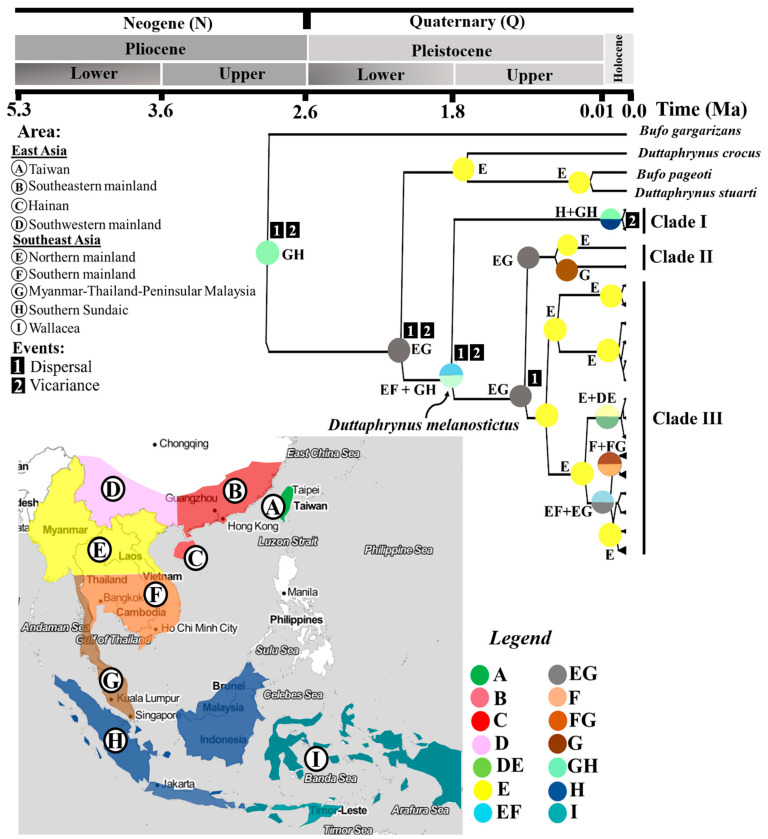
Ancestral range reconstruction and Statistical Dispersal Vicariance Analysis (S-DIVA) with dating estimates on clades divergence and inferred from mtDNA *tRNAGly ND3* fragment for 10 populations of 303 individuals *Duttaphrynus melanostictus*. A–H represent populations based on colour-coded geographical areas. EG (E + G), FG (F + G) and GH (G + H) represent the combined range of distribution for two areas. Missing geographical region in the tree nodes indicates anthropogenic introduction of this species in that part of the range.

Finally, we inferred the historical biogeography of *D. melanostictus* by performing a Statistical Dispersal-Vicariance Analysis (S-DIVA) and a Bayesian Binary MCMC (BBM) on the already calibrated Bayesian trees from the *tRNAGly–ND3* dataset using Range Ancestral State in Phylogeny (RASP v.4.0 [76]; Figure 3).

### 2.5. Estimating Past Population Dynamics

We tested for population expansions using Fu’s F test [77] and mismatch distribution pattern on the 334 bp of the *tRNAGly–ND3* mtDNA fragment using DNAsp v.5.0 [57] and we ran Tajima’s D test [78], Ramos-Onsins and Rozas’s R2 Test of Raggedness [79] using Arlequin v.3.5.2.2 [80] to conduct a neutrality test on population growth. Besides the statistical tests, we evaluated the impact of the LGM on past population dynamics of mainland populations of *D. melanostictus* (e.g., Southeast Asian and East Asian mainlands). We calculated the expansion rate of our mtDNA dataset with a Bayesian Coalescent Skyline Plot (BCSP; BEAST v.2.5.2; [81]). We determined five populations for further analyses based on clustering in the phylogenetic tree and the geographic landscape of the populations: “mainland population” refers to Southeast Asian mainland to East Asian mainland, continental Southeast Asia covers Southern Sundaic and Wallacea and continental Northeast Asia covers Taiwan. We ran the BCSP analysis for all five datasets separately in BEAST v.2.5.2 [81], including the totality of our dataset (*n* = 299) and four additional independent datasets: Southeast Asian mainland (*n* = 236), East Asia mainland (*n* = 51), Taiwan (*n* = 28) and Southern Sundaic-Wallacea (*n* = 15). Here, we also tested for the effects of the Sunda Shelf during the LGM on *D. melanostictus* on continental populations such as Southern Sundaic and Wallacea. Thus, based on landscape criterion and the phylogenetic inference showing clustering into clade I (Figure 2), we combined Southern Sundaic and Wallacea populations. For all designated populations independently, we selected the Coalescent Bayesian Skyline as ‘prior’ with GTR + Invariant + Gamma as site model, as this provided a good convergence of MCMC chains, with normal distribution parameter. We set a gamma category count to four with the four number of dimensions for both groups and populations in the settings. As our dataset consisted of a single species, we used a strict clock with the 0.049 clock rate calculated earlier. We ran the analysis for 1,000,000 generations of MCMC chains based on the diagnostic of good convergence value we obtained for all parameters, under the assumption that population dynamics are constant over time with no recombination. We diagnosed the convergence of the runs and generated the BCSP under standard default parameters through Tracer v.1.7.1 [75].

### 2.6. Genetic Admixtures Analyses

We estimated genetic admixture based on the 403 bp of the nuDNA fragment *SOX9* from a total of 126 homologous sequences (*n* collected in Taiwan = 10; *n* Genbank = 116; Table S1). First, we trimmed reverse and forward sequences to the same length with Champuru v.1.0 [82]. Next, to solve phase ambiguities resulting from heterozygosity, we checked the quality of reads with PHASE [83] using 1,000,000 MCMC chains run in DNASP v.5.0 [57]. For the subsequent analyses, we first segregated the 126 individuals into six independent populations based on both geographic region and landscape factors and resolved clades from the phylogenetic trees: Taiwan, East Asian mainland, Southeast Asian mainland, coastal Myanmar-Thailand-Peninsular Malaysia and Southern Sundaic with Wallacea. Using an admixture model and correlated allele frequencies assumption, we ran the Bayesian population structure computation without selection of sampling population criterion (Locprior = 0) with 150,000 step MCMC chains (10% burn-in) in STRUCTURE v.2.3.4 [84]. Admixture and Loc prior algorithms were selected here in order to test the probability of origin for all individuals, considering that either the entire population originated from a single origin, or that additional populations were located outside of the studied area. Based on the six populations (*n* = 6), we tested for the number of clusters (K) from 1 to 7 according to Evanno’s recommendation and conducted 15 replicates of the analysis, using the average value to determine the best K [85]. The value of K = 3 was selected after calculating delta K with Structure Harvester [86]. We then evaluated the best replicates in the determined cluster with 1000 randomized input replicates and selected the highest H’ value among the replicates using Clumpp [87]. We visualized the results using Structure Plot v.2.0 [88].

### 2.7. Isolation by Distance and Population Structure Analyses

To test the impact of anthropogenic activities on the distribution of *D. melanostictus*, we compared the genetic distance (F_ST_) between populations through isolation by distance (IBD) with a mantel test [89]. For this analysis, designated subpopulations were selected based on geographic isolation >100 km for mainland populations, and landscape resistance factor such as mountain ranges and sea channels for all continental populations [90]. Thus, our designated populations were: Taiwan, Southeastern China, Hainan, Southwestern China, Southeast Asian mainland, coastal Myanmar-Southern Thailand and Peninsular Malaysia, Southern Sundaic, Wallacea and Madagascar (refer to map in Figure 1). We then repeated the mantel test, excluding the invasive population in Madagascar. The Malagasy population was chosen as the outlier population in the model due to its geographic isolation. The aim of this analysis was to trace the ‘stepping stone’ route used by the invasive population in Madagascar. We conducted this analysis by comparing the value of IBD from the entire population versus the entire population minus the Malagasy one. In addition, we computed the diversity statistics using Arlequin ver3.5.2.2 [80] to assess genetic variation within designated populations.

## 3. Results

### 3.1. Haplotype Network

The statistical parsimony analysis based on the mtDNA *tRNAGly–ND3* fragment of *Duttaphrynus melanostictus* resulted in 67 distinct haplotypes, with 88 parsimonious sites. We identified three lineages (Figure 1): clade I comprised two haplotypes (*n* = 9), distributed from the continental population of Southeast Asia and including Southern Sundaic, Sumatra and Java to Wallacea, Sulawesi and Maluku. Clade II consisted of a single haplotype (*n* = 8), including populations from coastal Myanmar, Southern Thailand and Peninsular Malaysia. The largest and most diversified lineage was clade III, consisting of 64 haplotypes (*n* = 346) and including populations in Southeast Asian mainland (Northern Myanmar, Laos, Thailand, Cambodia, Vietnam), East Asian mainland (Southeastern China) and continental and islandic populations of East Asia (Hainan, Taiwan and invasive population in Madagascar; Figure 1, Table S1).

### 3.2. Phylogenetic Reconstruction Based on mtDNA Sequence

**Figure 4:**
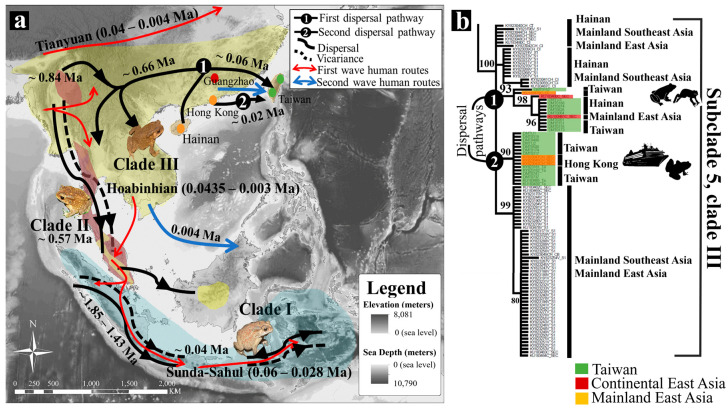
Hypothesised dispersal pathway of *Duttaphrynus melanostictus* in the Eastern Indomalayan realm. (**a**) The schematic diagram of predicted dispersal pathways for the three *D. melanostictus* lineages in the Indo Malayan realm integrating a model of prehistoric human dispersion from Southeast Asia during the Neolithic: Tianyuan (0.04–0.004 Ma), and Hoabinhian (0.0435–0.003 Ma) and Sunda-Sahul (0.06–0028 Ma); modified from Ji et al. (2016) [91], Yang et al. (2017) [92], Mccoll et al. (2018) [93], Gomes et al. (2015) [94], and the secondary human migration routes in Southern China post LGM adapted from Brandão et al. (2016) [95]. (**b**) Bayesian tree with an emphasis on the origin of contemporary population in Taiwan, either resulting from natural dispersion, or human mediated dispersion.

The topologies produced by the ML and BI trees were highly similar in regard to clade and branch topologies, with differences only found in posterior probabilities and support values (Figure 2). The phylogenetic tree for *tRNAGly–ND3* recovered three distinct monophyletic assemblage for *D. melanostictus*. Clade I was statistically supported, basal to the species’ clades topology and monophyletic (Bayesian Posterior Probability (BPP) = 100%, ML bootstraps support = 100/100). The clade comprised geographically isolated populations from the Southern Sundaic covering Sumatra, Java; and Wallacea covering Sulawesi and Maluku (Figure 2). Clade II formed a statistically supported monophyletic group (BPP = 100%, ML = 97/100) and included the populations from coastal areas in Southeast Asia ranging from Northern Myanmar, Southern Thailand to Peninsular Malaysia. Clade III formed a nested monophyletic group (BPP = 91%, ML = 94/100) and was divided into five subclades. Subclade 1 (BPP = 92%, ML = 98/100) was geographically overlapping with clade II in northern Myanmar, Southern Thailand and Peninsular Malaysia. Some individuals in subclade 1 were also present in Borneo and Western China (Figure 2). All four other subclades were also well supported, subclade 2: BPP = 92%, ML = 98/100, subclade 3: BPP = 100%, ML = 98/100, subclade 4: BPP = 30%, ML = 73/100 and subclade 5: BPP = 100%, ML = 94/100, encompassing all *D. melanostictus* distributed on the Southeast Asian mainland, East Asia mainland, Taiwan and the invasive population in Madagascar (Figure 2). The clustering of the Taiwanese clade showed that recent ancestors originated from populations in Southeastern China and Hong Kong (subclade 5; BPP = 94%, clade III, Figure 4b).

### 3.3. Molecular Dating and Ancestral Range Reconstruction

We dated the divergence time for the split between the genera *Duttaphrynus* and *Bufo* in Southeast Asia to 2.94 Ma (Standard deviation (SD) = 1.85–3.91 Ma; Figure 3, Table 1). The stem origin of *D. melanostictus* on the Southeast Asian mainland emerged around 1.84 Ma (SD = 1.01–2.74 Ma). *Duttaphrynus melanostictus* then became isolated through vicariance and dispersal in Southern Sundaic and Wallacea. Within *D. melanostictus*, the deepest split segregating Clade I is dated at 1.43 Ma (SD = 0.76–2.20 Ma). The split between clade II and clade III is dated at 0.84 Ma (SD = 0.45–1.30 Ma).

Later, populations from clade III dispersed from Northern Myanmar into the Southeast Asian mainland (Laos, Thailand, Cambodia, Vietnam) and the East Asian mainland (Southeastern China), with the two clades diverging 0.66 Ma (SD = 0.33–1.05 Ma). East of the Southeast Asian mainland was then colonised by clade II 0.59 Ma (SD = 0.26–0.92 Ma), explaining the geographical overlap between clade II and clade III in Myanmar. During the late Pleistocene, clade III diversified further, forming crown clades (0.35 Ma, SD = 0.15–0.56 Ma). Finally, clade I dispersed into Western Indonesia in the Holocene (0.04 Ma, SD = 13.98–0.013 Ma; Figure 3, Table 1), a population expansion that occurred much later than the colonisation of the Southeast Asian mainland by clade I and clade III. We summarize our understanding on the relative contribution of pre-historic human dispersion on the biogeography pattern of *D. melanostictus* in Indomalayan Realm in detail (Figure 4).

### 3.4. Population Dynamic over the Last Glacial Maximum

**Figure 5:**
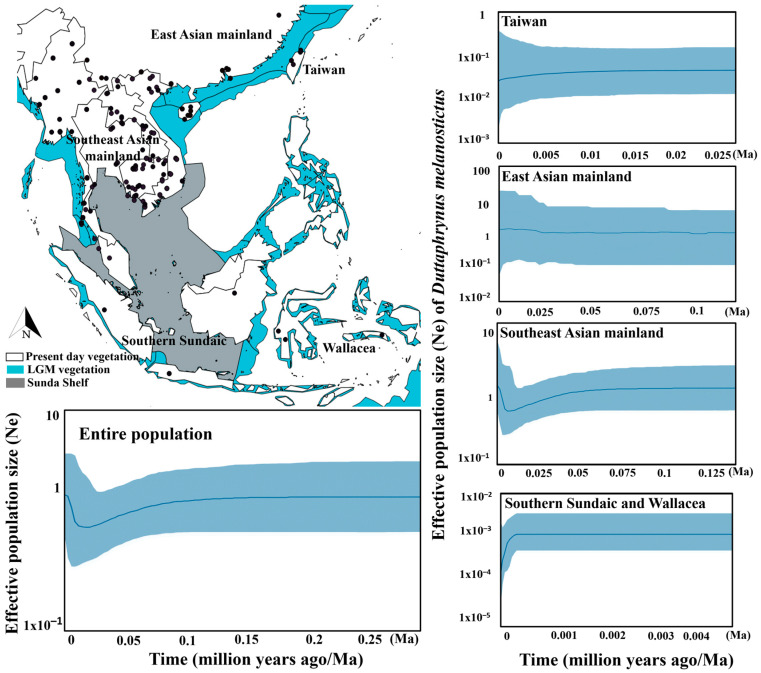
Bayesian Coalescent Skyline Plot analyses for all populations merged and selected population of interest for *Duttaphrynus melanostictus.* The blue line is the median estimate of the estimated effective population size. The shaded areas represent the 95% interval. The x-axis represents the time in years and y-axis represents the effective population size (Ne). The blue shade on the map indicates vegetation during LGM, with emphasis on Sunda shelf extension. The map was built using QGIS [96], overlaid with the LGM vegetation [97] and Sunda Shelf map [98].

The Bayesian Coalescent Skyline Plots (Figure 5) exhibiting population expansion of *D. melanostictus* since the LGM showed that the entire population of *D. melanostictus* on the Southeast Asian mainland expanded since the Anthropocene 0.0125 Ma (mean of likelihood = −42.17 ± 16.02, 95% HPD = −334.97 to 225.40; Figure 5). The population dynamic for Southeast Chinese mainland populations showed an expansion starting during the Holocene (0.125 Ma; mean of likelihood = −127.34 ± 15.35, 95% HPD = −94.24 to 338.95; Figure 5), concurrent with the gradual population expansions of *D. melanostictus* on the East Asian mainland and southern regions of the Chinese mainland (0.025 Ma to present; mean of likelihood = −712.24 ± 14.75, 95%, HPD = −798.32 to −50.89; Figure 5). In contrast, the Taiwanese population showed a decrease in population size over the same period (0.025 Ma to present; mean of likelihood = −45.69 ± 0.36, 95%, HPD = −70.39 to −21.17; Figure 5). The same negative population dynamic was recovered in other continental populations such as the Southern Sundaic and Wallacea for a similar period (0.004 Ma; mean of likelihood = 104.31 ± 2.14, 95% HPD = 87.25−122.51; Figure 5).

### 3.5. Population Cluster and Genetic Admixtures

**Figure 6:**
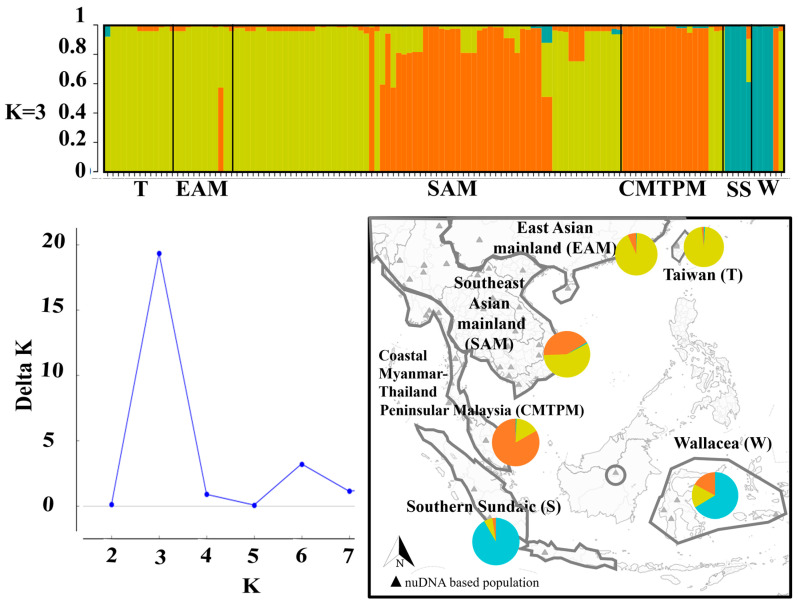
ADMIXTURE plot showing clustering by population for *Duttaphrynus melanostictus* inferred from SNP alleles from nuDNA *Sox9*. The number of independent populations is inferred through the calculation of Δ K [85]. On the bar plot, each column represents a single individual, and framed areas isolate each population such as defined on the map.

Bayesian population clustering resulted in three clusters as the most likely number of populations (*n* = 126; delta K = 19 when K = 3; Figure 6 and Table S2). The first group included the Southern Sundaic (*n* = 5, clade I; *p* = 0.917, clade III; *p* = 0.059, clade II; *p* = 0.024) and Wallacea (*n* = 6, clade I; *p* = 0.664, clade III; *p* = 0.165, clade II; *p* = 0.171), and demonstrated a characteristic of continental admixture as the genetic cluster is distributed restricted to the islands (Figure 6). The second group was principally present on the Southeast Asian mainland (*n* = 73, clade III; *p* = 0.572, clade II; *p* = 0.419, clade I; *p* = 0.008) and East Asian mainland (*n* = 11, clade III; *p* = 0.929, clade II; *p* = 0.067, clade I; *p* = 0.004), with equivalent bidirectional genetic mixtures between these two populations. The Taiwanese population (*n* = 12, clade III; *p* = 0.973; clade I; *p* = 0.009, clade II; *p* = 0.018) exhibited genetic admixture with mainland populations from Southeast Asia and East Asia (Figure 6 and Table S2). In contrast, the population in coastal Myanmar, Southern Thailand and Peninsular Malaysia (*n* = 19, clade II; *p* = 0.832, clade III; *p* = 0.163, clade I; *p* = 0.005; Figure 6 and Table S2) was distinguishable from the mainland Southeast Asian and East Asian populations, despite being connected by the same geographic landscape.

### 3.6. Population Genetics and Isolation by Distance

**Figure 7:**
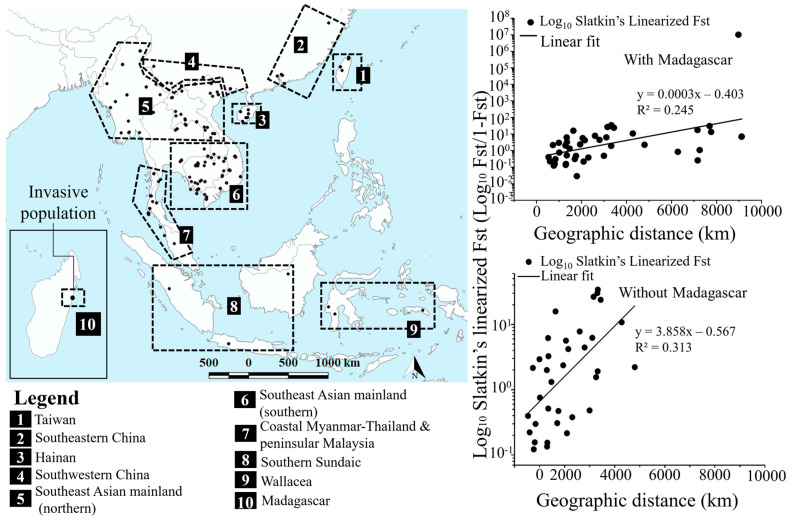
Comparative Isolation by Distance though mantel tests on ten versus nine populations, based on mtDNA *tRNAGly ND3* fragments. The scatter plots of Mantel Test for IBD show Slatkin’s linearized F_ST_ versus geographical distance across all localities of tested populations for *Duttaphrynus melanostictus*. The inclusion of the invasive population in Madagascar caused a noticeable decrease in pattern of genetic distance and the effectiveness of IBD correlation.

Out of the 10 populations tested, we found three populations with negative Fu’s Fs: Southeastern China (Fu’s F = −0.998; *p* < 0.005; Table 2); Hainan (Fu’s = −1.315; Table 2) and south of the Southeast Asian mainland (Fu’s = −17.476; *p* < 0.001; Table 2). Fu’s F tests were not significant for the seven other populations (*p* < 0.005; Table 2). Tajima’s D test was significant for seven populations, not including Southwestern China (Tajima’s D = −1.825; *p* = 0.013), south of the Southeast Asian mainland (Tajima’s D = −2.057; *p* = 0.003) and Southern Sundaic (Tajima’s D = −2.021; *p* = 0.001; Table 2). The Mantel test for IBD on the nine populations (i.e., excluding Madagascar; *n individuals* = 298) exhibited a significant IBD between populations (R^2^ = 0.313; *p* = 0.030; Figure 7), nucleotide diversity of 0.005 (±0.004) and pairwise differences of 1.643 (±1.078). Whereas, the IBD test on the 10 populations (including Madagascar; *n* individuals = 363) showed lower values of IBD (R^2^ = 0.245; *p* = 0.050; Figure 7) and a decreased pattern of genetic similarity between populations, with nucleotide diversity of 0.006 (±0.004) and pairwise differences of 2.111 (±1.221).

## 4. Discussion

The results of our phylogenetic reconstruction first confirmed the presence of three well supported lineages within *Duttaphrynus melanostictus* in the Eastern Indomalayan realm (Figure 2), in congruence with the comprehensive taxonomic revision by Wogan et al. (2016) [54]. Next, through mismatch distribution models we further document two lineages within the East Asian clade (Clade III, Table 2): one on the Southeast Asian mainland and one in Southeastern China (Table 2). We constrained the root of calibrated tree based on the context of the late phase of the Qinghai Tibetan uplift (QTP) that triggered the diversification of amphibians taxa distributed on the Asian mainland [99,100,101]. Thus, the calibrated treetime estimated that the common ancestor to the *D. melanostictus* lineage in Southeast Asia was younger than the Upper Neogene but older than the Lower Quaternary (Figure 3). Later, the divergence between lineages likely resulted from an expansion during the climatic transition between the warm climate of the Pliocene to the ice age of the Pleistocene (2.62–1.08 Ma; Figure 3, Table 1). Accordingly, the timing of dispersion onto the Southeast Asian mainland is consistent with the deep divergence between *D. melanostictus*, *Duttaphrynus* sp. and *D. parietalis*, distributed in the Western Ghats (Upper Pleistocene; 2.5 Ma; [102]).

### 4.1. Vicariance during the Lower Pleistocene

The biogeographical history inferred from the BBM tree and the S-DIVA reconstruction supports two mechanisms for the establishment of *D. melanostictus* in Southeast Asia. First, as a result of vicariance through isolation of clade I in the Southern Sundaic and Wallacea, and second through dispersal to the southern extent of Southeast Asia from current Myanmar during the Mid-Pleistocene Revolution (MPR, 1.53–1.33 Ma; referring to the crown node of clade I of *D. melanostictus* in Figure 3, Table 1).

The glaciation during the MPR resulted in the population being displaced from current Myanmar towards the coastal areas of Southern Thailand and Peninsular Malaysia, reaching as far south as Southern Sundaic and Wallacea as a result of lower sea levels (Figure 4a, Table 1). Thus, we determined that the most probable origin of the current three *D. melanostictus* lineages in Southeast Asia is likely from current Myanmar. This result is in agreement with the hypothesis that the most recent common ancestor of *D. melanostictus* diverged in the Western Ghats, current South Asia, before expanding to the Southeast Asia (Miocene; [102,103]). Several paleogeographic evidence on major avian and terrestrial fossils from Wallacea dated to the Mid to Upper Pleistocene highlight a population displacement pattern similar to the one described above for *D. melanostictus* [104].

Following genetic isolation, populations of *D. melanostictus* in Southern Sundaic and Wallacea are hypothesised to be fragmented and to show random effects of genetic drift, such as observed in Celebes Toad in the Wallacea (*Bufo celebensis*; [105]). In contrast, the insular population may have adaptively diverged from the mainland because of different ecological forces such as competition and predator pressures [43]. The same pattern is visible where different ecological requirements contributed to morphological and genetic variations, such as in the Morato’s Digger Toad, *Proceratophrys moratoi*, in the Southern Brazilian Cerrado. Similarly, the course of Tietê River resulted in ‘island-like’ populations with limited genetic exchange. As a result, populations adapted to different habitats and diverged from other populations further north [106].

### 4.2. Coastal-Mainland Lineages Split during MPR

Southeast Asian coastal populations in Myanmar, Southern Thailand and Peninsular Malaysia became isolated from the Southeast Asian mainland populations (stem node of clade II and clade III; Figure 3, Table 1) during the MPR (1.43–0.84 Ma). The glacial–interglacial cycles during the transition period resulted in environmental changes driving the isolation between the two clades [107]. This scenario is consistent with the biogeographic structure elicited by the MPR for other amphibian species on the Southeast Asian mainland. For instance, the diversification between Northern and Central lineages of treefrogs in Vietnam (*Rhacophorus* sp.; [108]) resulted from sea level fluctuation in the South China Sea [109] and variations in the Asian monsoon system [110].

We determined that the colonisation of coastal Myanmar, Southern Thailand and Peninsular Malaysia by clade II (0.53 Ma; see crown clade of Clade II in Figure 3; Figure 4a; Table 1) occurred before the isolation of clade III. Our results suggest that the emergence of oceanic shelves (MPR; 1.25–0.7 Ma; [111]) along with the cooling of climates [112] may have resulted in the local adaptation of clade II along the Southeast Asian coastline.

### 4.3. Population Expansion since the LGM

Clade III is now present on the Southeast and East Asian mainland and islands such as Hainan and Taiwan (Figure 3, Table 1). We present evidence of population extension on the mainland based on past positive population dynamics for *D. melanostictus* on the mainland (Figure 5). Here, we resolved a reciprocally monophyletic clade III that consisted of five subclades (Figure 2). The pattern of nested subclades in clade III also indicates the expansion of the clade in the Southeast mainland, promoted by the lack of barriers to gene flow in the lowland landscape (Mid-Pleistocene—LGM; 1.33–0.01 Ma).

The population of *D. melanostictus* distributed in Western Myanmar and Southwestern China shows a major split between subclades 1 and 2, and subclades 3, 4 and 5 (see subclades in clade III; Figure 2 and Figure 3). This result supports the idea that these two groups of sub-clades may be segregated by differences in landscape and altitude, consistent with the geological uplift of the Hengduan mountain in the Eastern Tibeto-Himalayan region that caused a dramatic elevational shift [113]. This uplifting has already been linked to sympatric speciation in other amphibians, such as *Amolops* in Myanmar [114], and the leaf litter toads, *Leptolalax purpurus* and *Leptolalax yingjiangensis* in Western Yunnan [115].

### 4.4. Land Bridges and Anthropogenic Dispersion

We pinpointed two geographic origins for the Taiwanese population. The oldest origin was traced back to the geographically close locality of Southeastern China as well as Hainan (Upper Pleistocene; 0.06 Ma; Figure 4b, Table 1). The second origin is contemporary and located in Hong Kong (Upper Pleistocene-Holocene; 0.02 Ma; Figure 4b, Table 1). The dual origins emphasise a joint effect of natural dispersal from the mainland followed by human-mediated dispersal from coastal hubs. The common ancestor to the Taiwanese and adjacent populations is dated prior to the geographical separation of Taiwan and the Asian mainland (Holocene; 0.01 Ma; [116]). The timing provides evidence that the land bridge connecting Taiwan to the Asian mainland during the LGM helped the dispersion of *D. melanostictus* to Taiwan [117]. 

In contrast to previous hypotheses that dissociated phylogenetic structure and anthropogenic-assisted dispersion of *D. melanostictus* [54], we found a pattern of secondary introduction of *D. melanostictus* in Taiwan from Hong-Kong (Upper Pleistocene-Holocene; 0.02 Ma; Figure 4b), presumably resulting from new human settlements (Neolithic; 0.015–0.006 Ma; Figure 4a; [95]). The geographical isolation of the island by the sea straight (about 50 m deep) prevented natural secondary colonisation of the island [118]. Thus, the clustering of haplotypes present in the geographically distant but trade-wise close Taiwan and Hong-Kong [119] suggests a recent human-induced introduction resulting in a secondary extension. This dispersion pathway for *D. melanostictus* is supported by the species resistance to drought [120] as exemplified by invasions in Eastern Wallacea [121] and Madagascar [122].

Along the same lines, the crown formation of clade I is much younger than that of crowns of clade II and clade III (Upper Pleistocene; 0.04 Ma; Figure 3, Figure 4a, Table 1). Its timing is compatible with the first human settlements in the Southern Sundaic, current Eastern Java, during the pre-Neolithic (Upper Pleistocene; 0.45 Ma; [94,123]). In agreement, the genetic admixture for the *SOX9* fragment (clade I in Figure 6) shows that gene flow was mostly from populations on the Southeast Asian mainland and towards Wallacea. This indicates that the recent expansion of *D. melanostictus* in Wallacea and the neighbouring islands such as Timor Leste is likely driven by anthropogenic activities. Our results corroborate those of other studies highlighting the invasive nature of *D. melanostictus* in the Wallacean archipelagos [121].

The negative population dynamics in the Southern Sundaic and Wallacea during the last interglacial illustrates that the Sunda shelf did not have a significant effect on the colonisation and isolation of clade I, as would have been expected from the fragmentation of the rainforest on the now submerged Sunda shelf (Upper Pleistocene; 0.05 Ma; [124]). The LGM paleoclimate shifted from cool to warm and caused a contraction of rain forests on the insular Southeast Asia, contributing to the isolation of *D. melanostictus* on core Southern Sundaic islands such as Sumatra and Java. At that period, the Southern Sundaic formed a transequatorial savannah corridor, thus restricting the gene flow from rainforest habitats to northern Southeast Asia [34].

The coupled impacts of the submergence of the Sunda shelf (LGM; 20,000–30,000 BP; [125]) and the rainforest contraction on Peninsular Malaysia during the last interglacial [126] resulted in a genetic barrier between Borneo and the Southern Sundaic. The subsequent anthropogenic activities [95] resulted in the introduction of clade III from the Southeast Asian mainland to Borneo.

### 4.5. IBD Outlier Population Resulting from Anthropologic Activities

*Duttaphrynus melanostictus* populations are under strong isolation by distance in Southeast Asia, showing the absence of gene flow between remote populations due to physical barriers. This supports the recurrent allopatric segregation in population structure [54]. Our results, however, highlighted one exception in relation to the pattern of contemporary gene flow. The addition of the Malagasy population resulted in a lowered IBD by reducing the regression value (Figure 7). This observed pattern of IBD shows that the introduction in Madagascar was followed by a substantial variation on local genetic variation, hinting at local adaptation and thus causing a greater concern [127]. The same impact of introduction was observed before in the American populations of the Southern Leopard frog (*Lithobates sphenocephalus*) where IBD was driven by a single outlying population in wetlands of Longleaf Pine Reserve. The outlying population caused a negative association between genetic diversity and wetland connectivity as one population that was supposed to be spatially segregated was in fact introduced [128].

## 5. Conclusions

In agreement with our hypotheses, the Quaternary glaciations were the main events that shaped the distribution of *Duttaphrynus melanostictus* in the Eastern Indomalayan realm. Vicariance and dispersal between Upper Pliocene to Lower Pleistocene were the consequences of glaciations and climatic transitions, resulting in the isolation of clade I on insular refugia in Southeast Asia, such as the Southern Sundaic and Wallacea (Mid-Pleistocene Revolution MPR; 1.85 to 1.0 Ma). Following the MPR, clades II and III diverged from each other around 0.84 Ma, following a restriction in gene flow and causing clade II to become isolated in stable habitats along coastal Myanmar, Southern Thailand and Peninsular Malaysia (Late MPR; 0.57 Ma). Meanwhile, populations from clade III dispersed over large areas and reached the Chinese mainland and islands such as Hainan and Taiwan (Upper Pleistocene; 0.06 Ma). Subsequent to natural dispersion, human-mediated dispersal resulted in population movements such as the secondary invasion of Taiwan (Holocene; 0.02 Ma) and the invasion of the Southern Sundaic and Wallacea (Upper Pleistocene; 0.04 Ma). While anthropogenic factors played a minor role in the population structure of *D. melanostictus*, they played an important role on recent invasion.

## Figures and Tables

**Table 1:** Summary of dating estimates, statistical dispersal-vicariance analysis and Bayesian Binary Method reconstruction analyses for *Duttaphrynus melanostictus* based on 303 sequences of mtDNA *tRNAGly-ND3* fragments, with a highlight on relevant nodes and route of dispersion.

Relevant Nodes	Dating Estimates (Mean/Ma ± SD)	Reconstruction of Ancestral State in Phylogenies Route	Events * (Matrix)		
			Vicariance	Dispersal	Extinction
Outgroup *Bufo gargarizans*	2.94 (±0.10)	Western China to Myanmar	1	2	0
Stem origin of *D. melanostictus*	1.85 (±0.77)	Myanmar to southern Sundaic (Sumatra) to Wallacea	3	4	0
Stem origin of *D. melanostictus* clade I	1.43 (±0.10)	Myanmar to southern Sundaic and Wallacea	2	3	0
Stem origin of *D. melanostictus* clade II and III	0.84 (±0.32)	Myanmar to eastern Mainland Southeast Asia	0	0	0
Crown origin of *D. melanostictus* complex I	0.04 (±13.94)	Wallacea to Southern Sundaic	1	2	0
Crown origin of *D. melanostictus* complex II	0.57 (±1.49)	Myanmar to Peninsular Malaysia	1	2	0
Crown origin of *D. melanostictus* complex III	0.66 (±0.67)	Myanmar to eastern Mainland Southeast Asia	0	0	0
Crown origin of Taiwanese population	0.06 (±5.96)	Southwestern China to Taiwan	0	0	0

**Table 2:** Population genetic analyses and test of population expansion on eight populations of *D. melanostictus* distributed in Indomalayan realm. The analyses consist of nucleotide diversity (π), haplotype diversity (Hd), Neutrality Test; Tajima’s D, Li and Fu statistic, Ramos-Onsins and Roza’s (R2), Raggedness (r) and mismatch distribution for *Duttaphrynus melanostictus* based on 363 sequences of mtDNA *tRNA*.

Population	Sample Size (No. of Haplotype)	Nucleotide Diversity (π)	Neutrality Tests				Ramos-Onsins and Rozas’s (R2)	Goodness-of Fit Tests	Mismatch Distribution
			Tajima’s D	*p* Value	Fu’s Fs	*p* Value		r (*p* Value)	
Taiwan	23 (3)	1.882	1.855	0.972	4.891	0.974	0.2353	0.7232	multimodal
Southeastern China	8 (4)	2.273	−0.033	0.536	−0.998	0.240	0.1572	0.0569	unimodal
Hainan	8 (5)	1.857	−0.168	0.458	−1.315	0.099	0.1779	0.0536	unimodal
Southwestern China	11 (5)	5.389	−1.825	0.013	4.742	0.974	0.2970	0.1667	multimodal
Northern Southeast Asian mainland	46 (13)	14.915	0.298	0.703	1.289	0.721	0.1007	0.0117	multimodal
Southern Southeast Asian mainland	36 (14)	1.431	−2.057	0.003	−17.476	0.000	0.0308	0.0474	unimodal
Coastal Myanmar, Thailand and Peninsular Malaysia	13 (6)	22.048	2.229	0.995	10.859	0.996	0.1991	0.0817	multimodal
Southern Sundaic	33 (2)	9.733	−2.021	0.001	7.916	1.000	0.2262	0.2937	multimodal
Wallacea	5 (1)	0.000	0.000	1.000	0.000	-	-	-	-
Madagascar	65 (1)	0.000	0.000	1.000	0.000	-	-	-	-

## Supplementary Materials

The following are available online at https://www.mdpi.com/2076-2615/10/7/1157/s1, Table S1: Sampling localities and haplotype information for all *Duttaphrynus melanostictus* samples used in this study; Table S2: DISTRUCT output for probability of population structure (K = 3) for 123 individuals in six designated populations based on SNP nuDNA *SOX9* fragment.
