# Supplementary material for: Impact of the Mid-Pleistocene Revolution and Anthropogenic Factors on the Dispersion of Asian Black-Spined Toads (Duttaphrynus melanostictus)
Source: Animals (Basel). 2020 Jul 8;10(7):1157. doi: 10.3390/ani10071157 (PMC7401666; doi:10.3390/ani10071157)
Supplement: Supplementary file 1 [file animals-10-01157-s001.pdf]

SUPPLEMENTARY MATERIALS

Table S1:

Sampling localities and haplotype information for all *Duttaphrynus melanostictus* samples used in this study.

Samples	Voucher	Locality	Latitude	Longitude	*tRNA-Gly-ND3*	*SOX9*	Reference	MtDNA Hap
DMTU1	*D. melanostictus1*	Tunghai University	24.178	120.604	MN339491	MN481437	This study	1
DMTU2	*D. melanostictus2*	Tunghai University	24.178	120.604	MN339492	-	This study	1
DMTU3	*D. melanostictus3*	Tunghai University	24.178	120.604	MN339486	MN481442	This study	51
0064	*D. melanostictus4*	Yangmingshan National Park	25.139	121.603	MN339489	-	This study	51
0067	*D. melanostictus5*	Yangmingshan National Park	25.139	121.603	-	MN481439	This study	1
0074	*D. melanostictus6*	Yangmingshan National Park	25.139	121.603	-	MN481438	This study	-
0151	*D. melanostictus7*	Yangmingshan National Park	25.139	121.603	MN339502	-	This study	51
0152	*D. melanostictus8*	Yangmingshan National Park	25.139	121.603	MN339498	MN481435	This study	49
0173	*D. melanostictus9*	Yangmingshan National Park	25.139	121.603	MN339500	-	This study	51
0174	*D. melanostictus10*	Yangmingshan National Park	25.139	121.603	MN339495	MN481436	This study	1
0188	*D. melanostictus11*	Yangmingshan National Park	25.139	121.603	MN339487	MN481433	This study	51
0201	*D. melanostictus12*	Yangmingshan National Park	25.139	121.603	MN339499	MN481440	This study	51
0239	*D. melanostictus13*	Yangmingshan National Park	25.139	121.603	-	-	This study	1
0316	*D. melanostictus14*	Yangmingshan National Park	25.139	121.603	MN339501	MN481441	This study	51
0317	*D. melanostictus15*	Yangmingshan National Park	25.139	121.603	MN339503	-	This study	1
0319	*D. melanostictus16*	Yangmingshan National Park	25.139	121.603	MN339490	-	This study	1
0490	*D. melanostictus17*	Yangmingshan National Park	25.139	121.603	MN339493	-	This study	1
0500	*D. melanostictus18*	Yangmingshan National Park	25.139	121.603	MN339494	-	This study	1
0737	*D. melanostictus19*	Yangmingshan National Park	25.139	121.603	MN339496	MN481434	This study	1
0741	*D. melanostictus20*	Yangmingshan National Park	25.139	121.603	MN339497	-	This study	1

0828	*D. melanostictus21*	Yangmingshan National Park	25.139	121.603	MN339485	-	This study	51
0874	*D. melanostictus22*	Yangmingshan National Park	25.139	121.603	MN339488	-	This study	51
	FMNH262751	Samling Logging Concession, near 12°16'45.7"N 106°56'36.4"E, 180m	12.279	106.943	KU183376	KU183214	Wogan et al. (2016) [1]	46
	FMNH257352	Siem Reap town <10m	13.374	104.844	KU183346	KU183189	Wogan et al. (2016) [1]	44
	LSUHC10119	Pursat Province, near O'Som	12.068	103.159	KY823036		Wogan et al. (2016) [1]	21
	LSUHC10120	Pursat Province, near O'Som	12.068	103.159	KY823037		Wogan et al. (2016) [1]	21
	LSUHC10558	Koh Rong, Broken Heart Guest House	10.677	103.261	KY823038		Wogan et al. (2016) [1]	22
SL15		Phnom Bokar	10.632	104.019	KY823034		Wogan et al. (2016) [1]	19
	FMNH263064	Kirirom National Park, near 11°19'14"N 104°04'56"E, 700m	11.321	104.082	KU183364	KU183204	Wogan et al. (2016) [1]	26
	FMNH263065	Kirirom National Park, near 11°19'14"N 104°04'56"E, 700m	11.321	104.082	KU183365	KU183205	Wogan et al. (2016) [1]	26
	FMNH263083	Cardamom Mountains, Phnom Chan Mountain, 11°26'30"N 103°47'00"E, 100-200m	11.442	103.783	KU183370	KU183210	Wogan et al. (2016) [1]	25
	FMNH263070	Bayon Temple, Angkor Wat complex, 13°26'30"N 103°51'46"E, <10m	13.442	103.863	KU183368	KU183208	Wogan et al. (2016) [1]	28
	LSUHC8564	Kampot Province, Bokor National Park, field station	10.626	104.025	KY823035		Wogan et al. (2016) [1]	28
	FMNH263059	Bokor National Park, SE Cardamom Mountains, near 10°37'35"N 104°01'30"E, 1000m	10.626	104.025	KU183362	KU183203	Wogan et al. (2016) [1]	28
	FMNH263055	Bokor National Park, SE Cardamom Mountains, 10°37'35"N 104°01'30"E, 1000m	10.626	104.025	KU183355	KU183196	Wogan et al. (2016) [1]	28
SL965		Phnom Penh	11.569	104.924	KY823033		Wogan et al. (2016) [1]	19
	FMNH262749	Virachey National Park, small tributary of O Lopeung stream, 14°11'16.3"N 107°17'36.1"E, 160m	14.188	107.293	KU183374	KU183213	Wogan et al. (2016) [1]	19

	FMNH262750	Virachey National Park, near 14°16'34.8"N 106°32'10.5"E, 200m	14.276	106.536	KU183375		Wogan et al. (2016) [1]	19
	FMNH262752	Samling Logging Concession, Keo Seima camp, 12°08'16.0"N 106°54'54.8"E, 160m	12.138	106.915	KU183377	KU183215	Wogan et al. (2016) [1]	19
	FMNH257354	Phnom Kulen Hill Range, 400m	13.567	104.145	KU183350	KU183193	Wogan et al. (2016) [1]	19
	FMNH263056	Bokor National Park, SE Cardamom Mountains, 10°37'35"N 104°01'30"E, 1000m	10.626	104.025	KU183356	KU183197	Wogan et al. (2016) [1]	19
	FMNH263054	Bokor National Park, SE Cardamom Mountains, 10°37'35"N 104°01'30"E, 1000m	10.626	104.025	KU183354	KU183195	Wogan et al. (2016) [1]	28
	FMNH263059	Bokor National Park, SE Cardamom Mountains, near 10°37'35"N 104°01'30"E, 1000m	10.626	104.025	KU183362	KU183203	Wogan et al. (2016) [1]	28
	FMNH263055	Bokor National Park, SE Cardamom Mountains, 10°37'35"N 104°01'30"E, 1000m	10.626	104.025	KU183355	KU183196	Wogan et al. (2016) [1]	28
K4941	MNHN 2010.7070	Tbeng Meanchey, Lac de barrage, Prae Vihear province, 50 m	13.77	104.951	KY823031		Vences et al. (2017) [2]	19
SL15		Phnom Bokar	11.569	104.924	KY823033		Vences et al. (2017) [2]	19
	FMNH262749	Virachey National Park, small tributary of O Lopeung stream, 14°11'16.3"N 107°17'36.1"E, 160m	14.188	107.293	KU183374	KU183213	Wogan et al. (2016) [1]	19
	FMNH262750	Virachey National Park, near 14°16'34.8"N 106°32'10.5"E, 200m	14.276	106.536	KU183375		Wogan et al. (2016) [1]	19
	FMNH257353	Kantrieng commune, Tatrow village,, <10m	13.339	104.102	KU183349	KU183192	Wogan et al. (2016) [1]	19
	FMNH263057	Bokor National Park, SE Cardamom Mountains, 10°37'35"N 104°01'30"E, 1000m	10.626	104.025	KU183357	KU183198	Wogan et al. (2016) [1]	19
	FMNH263056	Bokor National Park, SE Cardamom Mountains, 10°37'35"N 104°01'30"E, 1000m	10.626	104.025	KU183356	KU183197	Wogan et al. (2016) [1]	19
	FMNH257355	Siem Reap town, 13°22'25"N 103°50'38"E, <10m	13.374	103.844	KU183348	KU183191	Wogan et al. (2016) [1]	19

	FMNH263066	Kirirom National Park, near 11°19'14"N 104°04'56"E, 700m	11.321	104.082	KU183366	KU183206	Wogan et al. (2016) [1]	45
KIZ04739		Baiyun Mountain, Guangzhou City	23.202	113.303	KU183461	KU183295	Wogan et al. (2016) [1]	51
KIZ04715		Luofu Mountain, Lianhe Village, Zhengguo Town, Huizhou City	23.278	113.915	KU183460	KU183294	Wogan et al. (2016) [1]	50
MVZ230374		Hongzha River near Louma	19.134	109.957	KU183479	KU183312	Wogan et al. (2016) [1]	48
DM007		Hong Kong	22.41	114.12	KY823045		Vences et al. (2017) [2]	1
DM009		Hong Kong	22.41	114.12	KY823047		Vences et al. (2017) [2]	1
DM005		Hong Kong	22.41	114.12	KY823043		Vences et al. (2017) [2]	1
	KU311571	Shiwan Dashang Nature Reserve (Near town of Fulong)	21.846	107.889	KU183462	KU183296	Wogan et al. (2016) [1]	4
	KU311574	Town of Wang Le	21.857	107.862	KU183465	KU183299	Wogan et al. (2016) [1]	4
DM011			23.06	104.72	KY823049		Vences et al. (2017) [2]	3
	KU311573	Shiwan Dashang Nature Reserve	21.844	107.864	KU183464	KU183298	Wogan et al. (2016) [1]	2
DM003	ROM 44358	Kangle Hot Springs, creek below resort	18.702	109.661	KY823041		Vences et al. (2017) [2]	8
	LSUHC4107	Hainan Island, Diao Luo Shan	18.725	109.869	KY823055		Vences et al. (2017) [2]	8
	LSUHC4107	Diao Luo Shan	18.725	109.869	KU183467	KU183301	Wogan et al. (2016) [1]	8
DM001	ROM 44359	Bawaling Nature Reserve	19.36	109.039	KY823039		Vences et al. (2017) [2]	9
DM002	ROM 43075	Diao Luo Shan Forest Park	18.725	109.869	KY823040		Vences et al. (2017) [2]	7
DM006	ROM 28484	Hong Kong	22.41	114.12	KY823044		Vences et al. (2017) [2]	7
DM010	ROM 28488	Hong Kong	22.41	114.12	KY823048		Vences et al. (2017) [2]	7
DM008	ROM 28487	Hong Kong	22.41	114.12	KY823046		Vences et al. (2017) [2]	7

	MVZ230378	Hongzha River near Louma	19.134	109.957	KU183480	KU183313	Wogan et al. (2016) [1]	7
DM004	ROM 44357	on road to Jian Feng Ling Nature Reserve	18.309	109.289	KY823042		Vences et al. (2017) [2]	47
	ROM 41267	Simao, 12.3 km SE of Simao	23.06	104.72	KY823051		Vences et al. (2017) [2]	10
DM012	ROM 41267		23.06	104.72	KY823050		Vences et al. (2017) [2]	12
DM014		Simao, 8.8 km W of Simao	22.472	100.531	KY823052		Vences et al. (2017) [2]	12
		Simao, 8.8 km W of Simao	22.472	100.531	KY823053		Vences et al. (2017) [2]	12
DM016	ROM 41268	Simao, 8.8 km W of Simao	22.472	100.531	KY823054		Vences et al. (2017) [2]	12
	CAS234074	Longling County, Mengzhai River	24.239	99.0034	KU183436	KU183270	Wogan et al. (2016) [1]	13
	FMNH257944	east of Mekong River, near Ban Namon village, 19°04'39"N 102°08'44"E, 200-300m	19.078	102.146	KU183351	KU183194	Wogan et al. (2016) [1]	15
	FMNH271108	Nam Ha National Protected Area, Nam Maye Stream, near 20°52'08.0"N 101°03'19.0"E, 1000m elev.	20.869	101.055	KU183400	KU183234	Wogan et al. (2016) [1]	17
	FMNH257945	west of Mekong River, near 18°54'08"N 101°34'30"E, 250-400m	18.902	101.575	KU183352		Wogan et al. (2016) [1]	15
	NCSM76225	Sepon Mining Tenement	16.967	105.814	KU183402	KU183236	Wogan et al. (2016) [1]	19
	FMNH263072	Vientiane city, 17°58'41"N 102°39'16"E, 190m	17.978	102.654	KU183369	KU183209	Wogan et al. (2016) [1]	19
	FMNH255309	Dong Khanthung National Biodiversity Conservation Area, near Ban Nong Som Hong village, 14°17'N 105°40'E , 100m	14.283	105.667	KU183344		Wogan et al. (2016) [1]	19
	FMNH257953	Vientiane city, 17°58'41"N 102°39'16"E, 190m	17.978	102.654	KU183345	KU183188	Wogan et al. (2016) [1]	19
	FMNH257946	Vientiane city, 17°58'41"N 102°39'16"E, 190m	17.978	102.654	KU183353		Wogan et al. (2016) [1]	38
	NCSM76226	Sepon Mining Tenement	16.942	106.054	KU183403	KU183237	Wogan et al. (2016) [1]	43

	FMNH255314	Phou Hin Poun National Biodiversity Conservation Area, Ban Vieng village, 17°20'N 104°57'E, 200m	17.333	104.95	KU183347	KU183190	Wogan et al. (2016) [1]	41
	NCSM76224	Sepon Mining Tenement	16.959	106.05	KU183401	KU183235	Wogan et al. (2016) [1]	19
	FMNH257947	Phou Dendin National Biodiversity Conservation Area, near Nam Khang River, near 22°09'04"N 102°12'19"E, 600m	22.161	102.205	KU183358	KU183199	Wogan et al. (2016) [1]	12
	FMNH257949	Phou Dendin National Biodiversity Conservation Area, near Nam Khang River on Nam Kaw stream, near 22°09'39"N 102°11'07"E, 600m	22.161	102.185	KU183360	KU183201	Wogan et al. (2016) [1]	12
	FMNH257948	Phou Dendin National Biodiversity Conservation Area, near Nam Khang River on Nam Kaw stream, near 22°09'39"N 102°11'07"E, 600m	22.161	102.185	KU183359	KU183200	Wogan et al. (2016) [1]	12
S01		Ansarimasina	-0.003	49.3394	KY823060		Vences et al. (2017) [2]	40
S07		Ansarimasina	-18.2	49.3394	KY823066		Vences et al. (2017) [2]	46
S43		Ampasimagneva	-18.23	49.3425	KY823091		Vences et al. (2017) [2]	46
S03		Ansarimasina	-18.2	49.3394	KY823062		Vences et al. (2017) [2]	46
S42		Ampasimagneva	-18.23	49.3425	KY823090		Vences et al. (2017) [2]	46
S06		Ansarimasina	-18.2	49.3394	KY823065		Vences et al. (2017) [2]	46
S41		Ampasimagneva	-18.23	49.3425	KY823089		Vences et al. (2017) [2]	46
S08		Ansarimasina	-18.2	49.3394	KY823067		Vences et al. (2017) [2]	46
S41		Ampasimagneva	-18.23	49.3425	KY823088		Vences et al. (2017) [2]	46
S09		Ansarimasina	-18.2	49.3394	KY823068		Vences et al. (2017) [2]	46

S10		Ansarimasina	-18.2	49.3394	KY823069		Vences et al. (2017) [2]	46
S39		Ampasimagneva	-18.23	49.3425	KY823087		Vences et al. (2017) [2]	46
S38		Ampasimagneva	-18.23	49.3425	KY823086		Vences et al. (2017) [2]	46
S11		Ansarimasina	-18.2	49.3394	KY823070		Vences et al. (2017) [2]	46
S37		Ampasimagneva	-18.23	49.3425	KY823085		Vences et al. (2017) [2]	46
S12		Ansarimasina	-18.2	49.3394	KY823071		Vences et al. (2017) [2]	46
S36		Ampasimagneva	-18.23	49.3425	KY823084		Vences et al. (2017) [2]	46
S04		Ansarimasina	49.339	49.3394	KY823072		Vences et al. (2017) [2]	46
S14		Ansarimasina	-18.2	49.3394	KY823073		Vences et al. (2017) [2]	46
S15		Ansarimasina	-18.2	49.3394	KY823074		Vences et al. (2017) [2]	46
S35		Ampasimagneva	-18.23	49.3425	KY823083		Vences et al. (2017) [2]	46
S16		Ansarimasina	-18.2	49.3394	KY823075		Vences et al. (2017) [2]	46
S34		Ampasimagneva	-18.23	49.3425	KY823082		Vences et al. (2017) [2]	46
S18		Ansarimasina	-18.2	49.3394	KY823077		Vences et al. (2017) [2]	46
S19		Ansarimasina	-49.34	49.3394	KY823078		Vences et al. (2017) [2]	46
S20		Ampasimagneva	-18.23	49.3425	KY823079		Vences et al. (2017) [2]	46
S32		Ampasimagneva	-18.23	49.3425	KY823080		Vences et al. (2017) [2]	46
FRT1080		Unknown	-18.2	49.3394	KY823059		Vences et al. (2017) [2]	46
Dolch1		Unknown	-18.2	49.3394	KY823058		Vences et al. (2017) [2]	46

	S14	Ansarimasina	-18.2	49.3394	KU183373	KU183212	Wogan et al. (2016) [1]	19
	LSUHC7110	Pulau Langkawi	6.3667	99.8	KU183473	KU183307	Wogan et al. (2016) [1]	35
	LSUHC6718	Pulau Penang	5.3999	100.239	KU183472	KU183306	Wogan et al. (2016) [1]	40
	LSUHC6596	Cameron Highlands	4.4709	101.376	KU183471	KU183305	Wogan et al. (2016) [1]	40
	CAS235562	Kyaitong Township, Loi Mwe Region, Naung Cho village, border of Loi Mwe Wildlife Sanctuary, 21 12 27.1 N, 99 44 14.6 E.	17.047	96.1104	KU183437	KU183271	Wogan et al. (2016) [1]	15
	CAS235600	Mine Pyin Township, S of Mine Pyin Town, 21 20 14.5 N, 99 02 11.2 E	21.208	99.7374	KU183438	KU183272	Wogan et al., (2016)	13
DM018			23.8	120.8	KY823162		Vences et al., (2017)	1
DM017			23.8	120.8	KY823161		Vences et al., (2017)	1
		Mt. Chan-chu, Taipei City	25.009	121.544	KU183439	KU183273	Wogan et al., (2016)	1
		Mt. Chan-chu, Taipei City	25.009	121.544	KU183485	KU183317	Wogan et al., (2016)	1
K3036	K3036	Doi Chiang Dao, Chiang Mai province, 570 m	19.283	98.95	KY823163		Vences et al., (2017)	14
K922	P922	Sa Nang Manora Forest Park, Phang Nga province,	8.5106	98.5422	KY823165		Vences et al., (2017)	35
SL220		Phuket	7.97	98.39	KY823166		Vences et al., (2017)	36
	FMNH265901	Phu Wua Wildlife Sanctuary, park headquarters, 18°14'47.3"N 103°57'44.6"E, 175m	18.246	103.962	KU183383	KU183220	Wogan et al. (2016) [1]	19
	FMNH265906	Phu Jong-Na Yoi National Park, headquarters, 14°26’05.2"N 105°15’12.8"E, 230m	14.435	105.254	KU183386	KU183222	Wogan et al. (2016) [1]	19
	FMNH265909	Pang Si Da National Park, Pang Si Da waterfall, Nam Keo River, 13°59'42.9"N 102°12'23.3"E, 180m	13.995	102.206	KU183389	KU183224	Wogan et al. (2016) [1]	19

	FMNH265908	Pang Si Da National Park, headquarters, 13°58'58.1"N 102°12'16.6"E, 90m	13.983	102.205	KU183388	KU183223	Wogan et al. (2016) [1]	19
	FMNH265899	Phu Wua Wildlife Sanctuary, park headquarters, 18°14'47.3"N 103°57'44.6"E, 175m	18.246	103.962	KU183381		Wogan et al. (2016) [1]	19
	FMNH265896	Phu Luang Wildlife Sanctuary, Phu Luang Wildlife Research Station, 17°20’42.8"N 101°30’27.9"E, 940m	17.345	101.508	KU183378	KU183216	Wogan et al. (2016) [1]	19
	FMNH265911	Pang Si Da National Park, Pang Si Da waterfall, Nam Keo River, 13°59'42.9"N 102°12'23.3"E, 180m	13.995	102.206	KU183391	KU183225	Wogan et al. (2016) [1]	43
K922	P922	Sa Nang Manora Forest Park, Phang Nga province,	8.5106	98.5422	KY823165		Wogan et al. (2016) [1]	35
SL220		Phuket	7.97	98.39	KY823166		Wogan et al. (2016) [1]	36
	FMNH265901	Phu Wua Wildlife Sanctuary, park headquarters, 18°14'47.3"N 103°57'44.6"E, 175m	18.246	103.962	KU183383	KU183220	Wogan et al. (2016) [1]	19
	FMNH265909	Pang Si Da National Park, Pang Si Da waterfall, Nam Keo River, 13°59'42.9"N 102°12'23.3"E, 180m	13.995	102.206	KU183389	KU183224	Wogan et al. (2016) [1]	19
	FMNH265908	Pang Si Da National Park, headquarters, 13°58'58.1"N 102°12'16.6"E, 90m	13.983	102.205	KU183388	KU183223	Wogan et al. (2016) [1]	19
	FMNH265899	Phu Wua Wildlife Sanctuary, park headquarters, 18°14'47.3"N 103°57'44.6"E, 175m	18.246	103.962	KU183381		Wogan et al. (2016) [1]	19
	FMNH265896	Phu Luang Wildlife Sanctuary, Phu Luang Wildlife Research Station, 17°20’42.8"N 101°30’27.9"E, 940m	17.345	101.508	KU183378	KU183216	Wogan et al. (2016) [1]	19
	FMNH265911	Pang Si Da National Park, Pang Si Da waterfall, Nam Keo River, 13°59'42.9"N 102°12'23.3"E, 180m	13.995	102.206	KU183391	KU183225	Wogan et al. (2016) [1]	43
	FMNH265910	Pang Si Da National Park, Pang Si Da waterfall, Nam Keo River, 13°59'42.9"N 102°12'23.3"E, 180m	13.995	102.206	KU183390		Wogan et al. (2016) [1]	42

	FMNH268272	Ngao Falls National Park	9.9333	98.7167	KU183396	KU183230	Wogan et al. (2016) [1]	39
	FMNH257951	Phuket Marine Biological Center, on southern tip of Phuket Island, 07°48'02"N 98°24'34"E	7.8006	98.4094	KU183361	KU183202	Wogan et al. (2016) [1]	40
	FMNH268269	Kaeng Krung National Park	9.3	98.8667	KU183397	KU183231	Wogan et al. (2016) [1]	40
	FMNH265904	Phu Jong-Na Yoi National Park, headquarters, 14°26'05.2"N 105°15'12.8"E, 230m	14.435	105.254	KU183384	KU183221	Wogan et al. (2016) [1]	19
	FMNH268268	Huay Yang National Park	11.583	99.55	KU183392	KU183226	Wogan et al., (2016)	31
	FMNH268278	Huay Yang National Park	11.583	99.55	KU183393	KU183227	Wogan et al. (2016) [1]	31
	FMNH268283	Huay Yang National Park	11.583	99.55	KU183394	KU183228	Wogan et al. (2016) [1]	31
	FMNH265900	Phu Wua Wildlife Sanctuary, park headquarters, 18°14'47.3"N 103°57'44.6"E, 175m	18.246	103.962	KU183382	KU183219	Wogan et al. (2016) [1]	19
	FMNH265905	Phu Jong-Na Yoi National Park, headquarters, 14°26'05.2"N 105°15'12.8"E, 230m	14.435	105.254	KU183385		Wogan et al. (2016) [1]	34
	FMNH265907	Phu Jong-Na Yoi National Park, headquarters, 14°26'05.2"N 105°15'12.8"E, 230m	14.435	105.254	KU183387		Wogan et al. (2016) [1]	32
DM021	ROM 27207	Ba Be National Park	22.418	105.634	KY823190		Vences et al. (2017) [2]	4
DM024	ROM 27211	Ba Be National Park	22.418	105.634	KY823192		Vences et al. (2017) [2]	4
DM025	ROM 27212	Ba Be National Park	22.418	105.634	KY823193		Vences et al. (2017) [2]	4
		Tam Dao	21.454	105.644	KU183478	KU183311	Wogan et al., (2016)	4
K798	MNHN 2007.6238	Huu Lien, Longson Province, 70 m	21.506	106.345	KY823171		Vences et al. (2017) [2]	2
K799	MNHN 2007.6239	Huu Lien, Longson Province, 70 m	21.506	106.345	KY823172		Vences et al. (2017) [2]	2

DM020	ROM 27206	Ba Be National Park	22.418	105.634	KY823252		Vences et al. (2017) [2]	2
DM093	ROM 32521	Chi Linh vicinity; Hoang Hua Tham Village	21.212	106.478	KY823251		Vences et al. (2017) [2]	2
DM070	ROM 27175	Hanoi	21.033	105.817	KY823233		Vences et al. (2017) [2]	2
DM069	ROM 27174	Hanoi	21.033	105.817	KY823232		Vences et al. (2017) [2]	2
DM068	ROM 27173	Hanoi	21.033	105.817	KY823231		Vences et al. (2017) [2]	2
DM026	ROM 27203	Ba Be National Park	22.418	105.634	KY823194		Vences et al. (2017) [2]	2
DM022	ROM 27208	Ba Be National Park	22.418	105.634	KY823191		Vences et al. (2017) [2]	2
DM028	ROM 36188	Quang Thanh Village	22.629	105.913	KY823196		Vences et al. (2017) [2]	6
TZ 700-6		Ho-Chi Minh City Zoo	10.788	106.706	KY823183		Vences et al. (2017) [2]	46
TZ 700-10		Ho-Chi Minh City Zoo	10.788	106.706	KY823173		Vences et al. (2017) [2]	46
TZ 700-12		Ho-Chi Minh City Zoo	10.788	106.706	KY823175		Vences et al. (2017) [2]	46
TZ 700-11		Ho-Chi Minh City Zoo	10.788	106.706	KY823174		Vences et al. (2017) [2]	46
TZ 700-01		Ho-Chi Minh City Zoo	10.788	106.706	KY823178		Vences et al. (2017) [2]	46
TZ 700-04		Ho-Chi Minh City Zoo	10.788	106.706	KY823181		Vences et al. (2017) [2]	46
TZ 700-03		Ho-Chi Minh City Zoo	10.788	106.706	KY823180		Vences et al. (2017) [2]	46
TZ 700-09		Ho-Chi Minh City Zoo	10.788	106.706	KY823185		Vences et al. (2017) [2]	46
DM046	ROM 33857	Yok Don National Park; vicinity of Park HQ	12.854	107.813	KY823211		Vences et al. (2017) [2]	46
	FMNH259128	U Minh Thuong Nature Reserve, 09°32'40"N 105°05'11"E, <10m	9.5444	105.086	KU183371	KU183076	Wogan et al. (2016) [1]	19
DM044	ROM 33853	Yok Don National Park; vicinity of Park HQ	12.854	107.813	KY823209		Vences et al. (2017) [2]	19

DM053	ROM 37627	Cat Tien National Park	11.417	107.428	KY823217		Vences et al. (2017) [2]	19
DM062	ROM 28480	Tram Lap	14.44	108.383	KY823226		Vences et al. (2017) [2]	19
K3811	K3811	Hon Ba, Khank Hoa province, 65 m	12.132	109.033	KY823167		Vences et al. (2017) [2]	24
DM099		Hon Dat Mountain	10.105	104.894	KY823257		Vences et al. (2017) [2]	23
DM049	ROM 33856	Yok Don National Park; vicinity of Park HQ	12.854	107.813	KY823213		Vences et al. (2017) [2]	28
DM031	ROM 33887	Y+G140ok Don National Park; 2.5km SSW of park HQ	12.833	107.809	KY823199		Vences et al. (2017) [2]	30
DM033	ROM 32539	Yok Don National Park; 2.5km SSW of park HQ	12.833	107.809	KY823206		Vences et al. (2017) [2]	29
DM058	ROM 33163	Krong Pa	14.338	108.473	KY823222		Vences et al. (2017) [2]	28
DM036	ROM 33872	Yok Don National Park; 8 km W of Park HQ	12.853	107.793	KY823203		Vences et al. (2017) [2]	28
DM042	ROM 33868	Yok Don National Park; vicinity of Park HQ	12.854	107.813	KY823207		Vences et al. (2017) [2]	28
	DM039	ROM 33878	12.867	107.708	KY823205		Vences et al. (2017) [2]	19
TZ 700-14		Ho-Chi Minh City Zoo	10.788	106.706	KY823177		Vences et al. (2017) [2]	19
TZ 700-2		Ho-Chi Minh City Zoo	10.788	106.706	KY823179		Vences et al. (2017) [2]	19
TZ 700-13		Ho-Chi Minh City Zoo	10.788	106.706	KY823176		Vences et al. (2017) [2]	19
TZ 700-8		Ho-Chi Minh City Zoo	10.788	106.706	KY823184		Vences et al. (2017) [2]	19
TZ 700-9		Ho-Chi Minh City Zoo	10.788	106.706	KY823185		Vences et al. (2017) [2]	46
TZ Phu1		Phu Quoc	10.215	103.974	KY823186		Vences et al. (2017) [2]	19
TZ Phu2		Phu Quoc	10.215	103.974	KY823187		Vences et al. (2017) [2]	19
DM032	ROM 33855	Yok Don National Park; 2.5km SSW of park HQ	12.833	107.809	KY823200		Vences et al. (2017) [2]	19

DM045	ROM 33886	Yok Don National Park; vicinity of Park HQ	12.854	107.813	KY823210		Vences et al. (2017) [2]	19
DM047	ROM 33859	Yok Don National Park; vicinity of Park HQ	12.854	107.813	KY823212		Vences et al. (2017) [2]	19
DM052	ROM 37629	Cat Tien National Park	11.417	107.428	KY823216		Vences et al. (2017) [2]	19
DM050		Cat Tien National Park	11.417	107.428	KY823214		Vences et al. (2017) [2]	19
	FMNH259129	U Minh Thuong Nature Reserve, 09°35'45"N 105°06'36"E, <10m	9.5958	105.11	KU183372	KU183211	Vences et al. (2017) [2]	19
DM057	ROM 33166	Krong Pa	14.338	108.473	KY823221		Vences et al. (2017) [2]	20
DM061	ROM 28479	Tram Lap	14.44	108.383	KY823225		Vences et al. (2017) [2]	20
DM043	ROM 33861	Yok Don National Park; vicinity of Park HQ	12.854	107.813	KY823208		Vences et al. (2017) [2]	18
DM039	ROM 33878	Yok Don National Park; Dak Ken River Camp; forest path W of camp, N of Yok Don Mt.	12.867	107.708	KY823205		Vences et al. (2017) [2]	19
TZ 700-14		Ho-Chi Minh City Zoo	10.788	106.706	KY823177		Vences et al. (2017) [2]	19
TZ 700-13		Ho-Chi Minh City Zoo	10.788	106.706	KY823176		Vences et al. (2017) [2]	19
TZ 700-2		Ho-Chi Minh City Zoo	10.788	106.706	KY823179		Vences et al. (2017) [2]	19
TZ 700-8		Ho-Chi Minh City Zoo	10.788	106.706	KY823184		Vences et al. (2017) [2]	19
TZ Phu1		Phu Quoc	10.215	103.974	KY823186		Vences et al. (2017) [2]	19
TZ Phu2		Phu Quoc	10.215	103.974	KY823187		Vences et al. (2017) [2]	19
DM030	ROM 33875	Yok Don National Park; 2.5km SSW of park HQ	12.833	107.809	KY823198		Vences et al. (2017) [2]	19
DM029	ROM 33863	Yok Don National Park; 2.5km SSW of park HQ	12.833	107.809	KY823197		Vences et al. (2017) [2]	19
DM032	ROM 33855	Yok Don National Park; 2.5km SSW of park HQ	12.833	107.809	KY823200		Vences et al. (2017) [2]	19

DM035	ROM 32535	Yok Don National Park; 2.5km SSW of park HQ	12.833	107.809	KY823202		Vences et al. (2017) [2]	19
DM045	ROM 33886	Yok Don National Park; vicinity of Park HQ	12.854	107.813	KY823210		Vences et al. (2017) [2]	19
DM047	ROM 33859	Yok Don National Park; vicinity of Park HQ	12.854	107.813	KY823212		Vences et al. (2017) [2]	19
DM051	ROM 37630	Cat Tien National Park	11.417	107.428	KY823215		Vences et al. (2017) [2]	19
DM052	ROM 37629	Cat Tien National Park	11.417	107.428	KY823216		Vences et al. (2017) [2]	19
DM050		Cat Tien National Park	11.417	107.428	KY823214		Vences et al. (2017) [2]	19
	FMNH259129	U Minh Thuong Nature Reserve, 09°35'45"N 105°06'36"E, <10m	9.5958	105.11	KU183372	KU183211	Vences et al. (2017) [2]	19
DM057	ROM 33166	Krong Pa	14.338	108.473	KY823221		Vences et al. (2017) [2]	20
	ROM 28479	Tram Lap	14.44	108.383	KY823225		Vences et al. (2017) [2]	20
DM056	ROM 37625	Cat Tien National Park	11.417	107.428	KY823220		Vences et al. (2017) [2]	37
K453	MNHN 1999.5601	Sapa, main Camp, 1250 m	103,821	103,821	KY823168		Vences et al. (2017) [2]	11
K670	MNHN 1999.5603	Sapa, main Camp, 1250 m	22.33	103,821	KY823169		Vences et al. (2017) [2]	11
DM104	ROM 38090	Sa Pa vicinity	22.349	103.771	KY823262		Vences et al. (2017) [2]	12
DM103	ROM 38088	Sa Pa vicinity	22.349	103.771	KY823261		Vences et al. (2017) [2]	10
DM106	ROM 38087	Sa Pa vicinity	22.349	103.771	KY823263		Vences et al. (2017) [2]	10

Table S2:

DISTRUCT output for probability of population structure (K=3) for 123 individuals in six designated populations based on SNP nuDNA *SOX9* fragment. The data were generated by Clumpak program [3].

**Group**	**Individual**	**P1**	**P2**	**P3**
Taiwanese	0201-sox9-2_B	0.9222	0.0073	0.0704
Taiwanese	0316-SOX9-2_B	0.9896	0.0073	0.0031
Taiwanese	0067-sox9-2_B	0.9897	0.0073	0.003
Taiwanese	DMTU3-SOX9-2_B	0.9898	0.0072	0.003
Taiwanese	0174-sox9-2_B	0.9884	0.0084	0.0031
Taiwanese	DMTU1-SOX9-2_B	0.989	0.008	0.003
Taiwanese	0737-SOX9-2_B	0.9589	0.0371	0.004
Taiwanese	0152-sox9-2_B	0.9587	0.0373	0.004
Taiwanese	0188-sox9-2_B	0.958	0.038	0.004
Taiwanese	0074-dmsox9-2_B	0.9596	0.0364	0.004
Taiwanese	KU183273.1-2_B	0.9846	0.0119	0.0036
Taiwanese	KU183317.1-2_B	0.9889	0.0081	0.003
Chinese	KU183298.1-2_B	0.9579	0.0381	0.004
Chinese	KU183270.1-2_B	0.9591	0.0369	0.004
Chinese	KU183312.1-2_B	0.9604	0.0356	0.004
Chinese	KU183294.1-2_B	0.9856	0.0112	0.0032
Chinese	KU183313.1-2_B	0.9892	0.0078	0.003
Chinese	KU183299.1-2_B	0.9896	0.0074	0.003
Chinese	KU183301.1-2_B	0.9897	0.0073	0.003
Chinese	KU183296.1-2_B	0.9908	0.0062	0.003
Chinese	KU183295.1-2_B	0.986	0.0107	0.0033
Chinese	KU183310.1-2_B	0.4231	0.5716	0.0053
Chinese	KU183297.1-2_B	0.986	0.0107	0.0033
Indochinese	KU183236.1-2_B	0.9591	0.0369	0.004
Indochinese	KU183229.1-2_B	0.9811	0.0149	0.004
Indochinese	KU183230.1-2_B	0.9813	0.0149	0.0038
Indochinese	KU183210.1-2_B	0.9598	0.0362	0.004
Indochinese	KU183221.1-2_B	0.958	0.038	0.004
Indochinese	KU183209.1-2_B	0.9822	0.0138	0.004
Indochinese	KU183206.1-2_B	0.9823	0.0138	0.0039
Indochinese	KU183215.1-2_B	0.9591	0.0369	0.004
Indochinese	KU183214.1-2_B	0.9586	0.0374	0.004
Indochinese	KU183213.1-2_B	0.9606	0.0354	0.004
Indochinese	KU183235.1-2_B	0.9593	0.0367	0.004
Indochinese	KU183207.1-2_B	0.9585	0.0375	0.004

Indochinese	KU183311.1-2_B	0.9594	0.0366	0.004
Indochinese	KU183203.1-2_B	0.9595	0.0367	0.0038
Indochinese	KU183208.1-2_B	0.9599	0.0361	0.004
Indochinese	KU183226.1-2_B	0.9589	0.0372	0.0039
Indochinese	KU183200.1-2_B	0.9894	0.0076	0.003
Indochinese	KU183234.1-2_B	0.9859	0.0107	0.0034
Indochinese	KU183202.1-2_B	0.9889	0.0081	0.003
Indochinese	KU183219.1-2_B	0.9886	0.0084	0.003
Indochinese	KU183220.1-2_B	0.9841	0.0122	0.0037
Indochinese	KU183222.1-2_B	0.9847	0.0121	0.0032
Indochinese	KU183218.1-2_B	0.9881	0.0084	0.0034
Indochinese	KU183211.1-2_B	0.9862	0.01	0.0038
Indochinese	KU183224.1-2_B	0.9611	0.035	0.0039
Indochinese	KU183212.1-2_B	0.9431	0.0484	0.0086
Indochinese	KU183271.1-2_B	0.0101	0.9869	0.003
Indochinese	KU183239.1-2_B	0.9572	0.0388	0.004
Indochinese	KU183225.1-2_B	0.3995	0.5964	0.0041
Indochinese	KU183237.1-2_B	0.0537	0.9422	0.0041
Indochinese	KU183232.1-2_B	0.4214	0.5734	0.0052
Indochinese	KU183201.1-2_B	0.1829	0.8122	0.0049
Indochinese	KU183228.1-2_B	0.1902	0.8016	0.0082
Indochinese	KU183264.1-2_B	0.1859	0.8095	0.0047
Indochinese	KU183245.1-2_B	0.1813	0.8139	0.0048
Indochinese	KU183250.1-2_B	0.1778	0.8175	0.0048
Indochinese	KU183223.1-2_B	0.01	0.987	0.003
Indochinese	KU183290.1-2_B	0.01	0.987	0.003
Indochinese	KU183256.1-2_B	0.0066	0.9914	0.002
Indochinese	KU183268.1-2_B	0.0245	0.9723	0.0032
Indochinese	KU183282.1-2_B	0.0262	0.9703	0.0034
Indochinese	KU183253.1-2_B	0.0252	0.9712	0.0036
Indochinese	KU183265.1-2_B	0.0245	0.9724	0.0031
Indochinese	KU183251.1-2_B	0.1849	0.8102	0.0049
Indochinese	KU183260.1-2_B	0.183	0.8125	0.0046
Indochinese	KU183272.1-2_B	0.1844	0.811	0.0046
Indochinese	KU183257.1-2_B	0.0323	0.9638	0.0039
Indochinese	KU183284.1-2_B	0.0189	0.9776	0.0036
Indochinese	KU183283.1-2_B	0.008	0.9894	0.0026
Indochinese	KU183258.1-2_B	0.0108	0.9862	0.003
Indochinese	KU183266.1-2_B	0.011	0.986	0.003
Indochinese	KU183262.1-2_B	0.0832	0.9125	0.0042
Indochinese	KU183267.1-2_B	0.0832	0.9128	0.004
Indochinese	KU183289.1-2_B	0.1818	0.8132	0.005
Indochinese	KU183227.1-2_B	0.025	0.9715	0.0034
Indochinese	KU183252.1-2_B	0.027	0.9697	0.0033
Indochinese	KU183243.1-2_B	0.0071	0.9788	0.0141
Indochinese	KU183249.1-2_B	0.0104	0.9776	0.012

Indochinese	KU183205.1-2_B	0.3679	0.5127	0.1194
Indochinese	KU183247.1-2_B	0.3697	0.5098	0.1204
Indochinese	KU183248.1-2_B	0.9609	0.0352	0.0039
Indochinese	KU183240.1-2_B	0.9588	0.0373	0.0039
Indochinese	KU183287.1-2_B	0.9532	0.0399	0.0069
Indochinese	KU183242.1-2_B	0.7521	0.2439	0.004
Indochinese	KU183263.1-2_B	0.7546	0.2414	0.004
Indochinese	KU183259.1-2_B	0.7536	0.2423	0.0041
Indochinese	KU183241.1-2_B	0.9606	0.0354	0.004
Indochinese	KU183291.1-2_B	0.9593	0.0367	0.004
Indochinese	KU183244.1-2_B	0.959	0.037	0.004
Indochinese	KU183255.1-2_B	0.9586	0.0373	0.0041
Indochinese	KU183231.1-2_B	0.9585	0.0375	0.004
Indochinese	KU183233.1-2_B	0.9361	0.0285	0.0355
Indochinese	KU183204.1-2_B	0.9346	0.0293	0.0361
Southern Peninsula	KU183293.1-2_B	0.0077	0.9896	0.0028
Southern Peninsula	KU183254.1-2_B	0.0057	0.9923	0.002
Southern Peninsula	KU183261.1-2_B	0.0059	0.992	0.0021
Southern Peninsula	KU183306.1-2_B	0.0058	0.9922	0.002
Southern Peninsula	KU183285.1-2_B	0.006	0.992	0.002
Southern Peninsula	KU183246.1-2_B	0.0162	0.9807	0.0031
Southern Peninsula	KU183292.1-2_B	0.0154	0.9816	0.003
Southern Peninsula	KU183281.1-2_B	0.016	0.981	0.003
Southern Peninsula	KU183309.1-2_B	0.0097	0.9873	0.003
Southern Peninsula	KU183286.1-2_B	0.0072	0.9902	0.0026
Southern Peninsula	KU183288.1-2_B	0.0102	0.9781	0.0117
Southern Peninsula	KU183269.1-2_B	0.0104	0.9773	0.0122
Southern Peninsula	KU183300.1-2_B	0.0497	0.9461	0.0042
Southern Peninsula	KU183302.1-2_B	0.0102	0.9777	0.0121
Southern Peninsula	KU183303.1-2_B	0.0101	0.978	0.0119
Southern Peninsula	KU183304.1-2_B	0.0101	0.9778	0.0121
Southern Peninsula	KU183307.1-2_B	0.9848	0.0117	0.0036
Southern Peninsula	KU183305.1-2_B	0.9571	0.0389	0.004
Southern Peninsula	KU183308.1-2_B	0.961	0.035	0.004
Sundanese	KU183275.1-2_B	0.0037	0.003	0.9933
Sundanese	KU183277.1-2_B	0.0038	0.003	0.9932
Sundanese	KU183276.1-2_B	0.003	0.0022	0.9948
Sundanese	KU183315.1-2_B	0.0051	0.004	0.9909
Sundanese	KU183316.1-2_B	0.2927	0.0967	0.6106
Walacean	KU183280.1-2_B	0.0046	0.0038	0.9917
Walacean	KU183314.1-2_B	0.003	0.003	0.994
Walacean	KU183279.1-2_B	0.0052	0.0041	0.9907
Walacean	KU183278.1-2_B	0.005	0.004	0.991
Walacean	KU183238.1-2_B	0.0104	0.9777	0.0119
Walacean	KU183274.1-2_B	0.9594	0.0366	0.004

References:

1. Wogan, G.O.U.; Stuart, B.L.; Iskandar, D.T.; McGuire, J.A. Deep genetic structure and ecological divergence in a widespread human commensal toad. *Biology Letters* **2016**, *12*, 20150807, doi:10.1098/rsbl.2015.0807.
2. Vences, M.; Brown, J.L.; Lathrop, A.; Rosa, G.M.; Cameron, A.; Crottini, A.; Dolch, R.; Edmonds, D.; Freeman, K.L.M.; Glaw, F.; et al. Tracing a toad invasion: lack of mitochondrial DNA variation, haplotype origins, and potential distribution of introduced *Duttaphrynus melanostictus* in Madagascar. *Amphibia-Reptilia* **2017**, *38*, 197–207, doi:10.1163/15685381-00003104.
3. Kopelman, N.M.; Mayzel, J.; Jakobsson, M.; Rosenberg, N.A.; Mayrose, I. CLUMPAK: a program for identifying clustering modes and packaging population structure inferences across K. *Molecular Ecology Resource* **2015**, *15*, 1179–1191, doi:10.1002/cncr.27633.Percutaneous.
